# LC-PUFA Biosynthesis in Aquatic Animals: A Hybrid Bibliometric and Thematic Review

**DOI:** 10.3390/biology15201840

**Published:** 2026-10-14

**Authors:** Faryal Gohar, Bihui Chao, Yongcheng Xue, Ye Yuan, Mhd Ikhwanuddin, Hongyu Ma

**Affiliations:** 1 Guangdong Provincial Key Laboratory of Marine Biotechnology, Shantou University, Shantou 515063, China; 2 International Joint Research Center for the Development and Utilization of Important Mariculture Varieties Surrounding the South China Sea Region, Shantou University, Shantou 515063, China; 3 STU-UMT Joint Shellfish Research Laboratory, Shantou University, Shantou 515063, China; 4 Agro-Tech Extension Center of Guangdong Province, Guangzhou 511499, China; 5 Higher Institute Centre of Excellence (HICoE), Institute of Tropical Aquaculture and Fisheries, Universiti Malaysia Terengganu, Kuala Nerus 21030, Malaysia

**Keywords:** LC-PUFA biosynthesis, aquatic animals, bibliometric analysis, trophic transfer, aquaculture

## Abstract

Long-chain polyunsaturated fatty acids (LC-PUFAs) are essential to the nutrition, metabolism, and physiological performance of aquatic animals. This hybrid review combines bibliometric analysis with focused thematic synthesis to examine three decades (1991–2025) of research on LC-PUFA biosynthesis in aquatic species. Bibliometric trends reveal rising publication output and an expanding research focus on biosynthetic pathways, regulatory mechanisms, and molecular control of LC-PUFA production, with emerging emphasis on metabolomics, environmental regulation, microalgae-derived ω-3 sources, and gene-editing technologies. Thematic analysis reveals that LC-PUFA dynamics are governed by interactions among primary production, trophic transfer, endogenous biosynthetic capacity, and environmental conditions. Marine primary producers serve as major sources of EPA and DHA, whereas freshwater and terrestrial systems often exhibit greater ALA availability and higher n-6/n-3 ratios, influencing LC-PUFA transfer efficiency and consumer adaptation. The review further highlights that aquatic animals have evolved diverse endogenous biosynthetic strategies, including lineage-specific modifications of desaturase and elongase pathways, enabling adaptation to contrasting nutritional environments. LC-PUFA transport across aquatic–terrestrial boundaries through organisms such as emergent insects and amphibians represents an important pathway for transferring essential fatty acids to terrestrial food webs. Furthermore, variation in n-3 LC-PUFA availability contributes to habitat selection and evolutionary diversification, with freshwater environments promoting enhanced biosynthetic capacity through mechanisms such as *Fads2* gene duplication. Future directions include structural characterization of biosynthetic enzymes via cryo-electron microscopy and AI-assisted modeling, multi-omics and genome-editing strategies for genetic improvement, and sustainable production approaches including microalgae- and insect-based feeds and integrated multitrophic aquaculture.

## Simple Summary

Long-chain polyunsaturated fatty acids are important fats that support the growth, development, health, and reproduction of aquatic animals and are also valuable nutrients for humans. Their availability depends on natural food sources, transfer through food webs, the ability of aquatic animals to produce them, and environmental conditions. This review examined 1083 scientific publications from 1991 to 2025 to understand how research in this field has developed. The findings show that research activity has increased considerably and that small aquatic organisms, particularly microalgae, are major natural sources of these fats. Their transfer through food webs influences the nutritional quality of aquatic animals, while the ability to produce them varies among species and has evolved in response to different diets and environments. Understanding these differences and the factors controlling fatty acid production can guide future research and support the nutritional quality and sustainability of aquatic food resources.

## 1. Introduction

Long-chain polyunsaturated fatty acids (LC-PUFAs) are fatty acids containing 20 or more carbon atoms and two or more double bonds. Major LC-PUFAs include arachidonic acid (ARA, 20:4n-6) of the n-6 series and eicosapentaenoic acid (EPA, 20:5n-3) and docosahexaenoic acid (DHA, 22:6n-3) of the n-3 series [1]. In aquatic ecosystems, EPA and DHA are synthesized de novo by diverse microorganisms, including certain microalgae, protists, fungi, and bacteria [2]. Several marine bacterial taxa are also capable of synthesizing LC-PUFAs, including EPA and DHA, through specialized PUFA biosynthetic pathways [3]. Terrestrial animals, including humans, cannot biosynthesize n-3 LC-PUFAs to achieve optimal cellular function [4]. Therefore, dietary intake of seafood, especially fish and shellfish, is the principal means by which humans acquire EPA and DHA [5,6]. The LC-PUFA content of fishery products varies according to species, habitat, geographic origin, and time of harvest [7]. Beyond their importance for human health, n-3 LC-PUFAs are important dietary nutrients that support normal growth, development, and physiological functions in fish, although their requirements vary among species and developmental stages [8,9].

LC-PUFAs, particularly EPA and DHA, also have important physiological functions in humans. EPA and DHA contribute to cardiovascular function and immune regulation, while DHA is particularly important for neural and retinal development [10]. Together with their essential physiological roles in aquatic animals, these health benefits emphasize the nutritional importance of maintaining adequate LC-PUFA availability.

The accumulation of n-3 LC-PUFAs in fish results from both dietary intake and endogenous biosynthesis [11]. In fish, the endogenous biosynthesis of LC-PUFAs from C18 polyunsaturated fatty acid (PUFA) precursors generally proceeds through sequential desaturation and elongation reactions, with biosynthetic capacity varying among species according to the presence, expression, and activity of key enzymes, particularly fatty acyl desaturases (Fads) and elongases of very long-chain fatty acids (Elovl) [12,13]. Vertebrates cannot synthesize the C18 essential fatty acids, namely, linoleic acid (LIN, 18:2n-6) and α-linolenic acid (ALA, 18:3n-3) de novo because they lack the required methyl-end desaturases; therefore, these precursors must be obtained from the diet [14]. Dietary LIN and ALA can subsequently be converted to n-6 and n-3 LC-PUFAs, respectively, although the efficiency of these pathways varies considerably among species. Consequently, species with limited endogenous LC-PUFA biosynthetic capacity depend more strongly on dietary sources of preformed LC-PUFAs, particularly EPA and DHA, which in conventional aquafeeds are supplied mainly by fish oil and, to a lesser extent, fishmeal [15,16]. In contrast to vertebrates, methyl-end desaturases are widely distributed among invertebrate taxa, including cnidarians, nematodes, rotifers, annelids, mollusks, and arthropods [17].

The enzyme complex fatty acid synthase generates the C16 saturated FA palmitic acid (16:0), whereas elongation enzymes of the Elovl family can elongate it to stearic acid (18:0) [18]. These saturated FAs can subsequently undergo desaturation at different positions to generate a range of monounsaturated FAs. A common pathway involves Δ9 desaturation, producing palmitoleic acid (16:1n-7) from 16:0 and oleic acid (18:1n-9) from 18:0; however, other desaturation positions can generate alternative isomers, including 16:1n-9, 16:1n-6, 16:1n-5, 18:1n-7, and 18:1n-11 [18,19]. The introduction of an additional double bond into monounsaturated FAs is an important step in de novo PUFA biosynthesis. A methyl-end desaturase with Δ12 activity converts oleic acid into LIN, which can subsequently be further desaturated by a Δ15 desaturase to produce ALA [20].

Methyl-end desaturases, including Δ12 and Δ15 desaturases, are membrane-associated enzymes that insert new double bonds toward the methyl (CH_3_) end of FAs. While such enzymes are present in plants and microorganisms, animals were initially thought to lack them, a perception later challenged by their identification in *Caenorhabditis elegans* [21]. This has been further revised following recent discoveries showing that many invertebrates also have these enzymes [17]. Subsequent functional analyses have revealed that animal methyl-end desaturases are capable of catalyzing both Δ12 and Δ15 reactions [22]. Methyl-end desaturases introduce double bonds at positions defined relative to the methyl terminus of FAs. ω-3 desaturases convert n-6 PUFAs into their corresponding n-3 products and can exhibit Δ15, Δ17, or Δ19 activities depending on whether they act on C18, C20, or C22 substrates, respectively [17]. In contrast, ω-6 desaturase activity introduces a double bond at the ω-6 position; for example, Δ12 desaturation of oleic acid (18:1n-9) produces LIN. In the diatom *Phaeodactylum tricornutum*, a Δ12-desaturase has also been shown to desaturate both 18:1n-9 and 20:1n-9, producing 18:2n-6 and 20:2n-6, respectively [23].

The availability and sustainable production of LC-PUFAs for human nutrition and aquaculture can be enhanced through several complementary approaches, including (1) genetic engineering of terrestrial oilseed crops through the introduction of LC-PUFA biosynthetic genes; (2) biotechnological production using microorganisms; and (3) aquaculture-based production systems [2]. Elucidating the mechanisms and regulation of LC-PUFA biosynthesis is important for understanding the nutritional physiology of aquatic animals and for supporting sustainable aquaculture. However, the increasing volume of literature and the diversity of studies across aquatic taxa, biosynthetic pathways, regulatory mechanisms, and ecological contexts make it difficult to systematically identify major research trends, influential contributors, collaborative patterns, and emerging topics using conventional narrative assessment alone. Bibliometric analysis provides a quantitative and systematic approach to examining these patterns within a large body of scientific literature.

Bibliometric analysis is widely used to provide a quantitative approach for examining publication patterns, research trends, collaboration networks, and the development of a scientific field [24,25]. Various databases can be used in bibliometric analyses, each with specific features and services [26]. This method improves the assessment of relationships among journal citations and goes beyond conventional approaches to citation impact analysis [27,28]. Despite its practicality, bibliometric analysis has not yet been applied to review the research on LC-PUFA production in aquatic species. In this review, we conducted a detailed bibliometric overview of LC-PUFA biosynthesis research in aquatic animals over the past 35 years (1991–2025) to identify research trends, key contributors, and emerging research directions in the field. Our bibliometric assessment included annual publication trends, country- and institution-wise contributions, the most productive and influential authors, leading journals, and citation patterns. We also analyzed collaborative dynamics through co-authorship networks, international partnerships, and keyword co-occurrence to provide an overview of the structure and evolution of the research landscape.

In addition to a bibliometric analysis, we present a thematic review of LC-PUFA research in aquatic systems, integrating multiple biological and ecological perspectives. The review first highlights the central role of microalgae as primary producers, emphasizing their diversity in LC-PUFA composition and the trophic transfer of LC-PUFAs through aquatic ecosystems. Building on this, we examine LC-PUFA biosynthesis from biochemical and evolutionary perspectives, including the canonical biosynthetic pathways characterized in fish and the diversification of endogenous LC-PUFA production across aquatic animals. We further explore how metabolic plasticity, variation in gene repertoires, and ecological pressures have shaped species-specific capacities for LC-PUFA synthesis. Beyond individual organisms, we assess the distribution and movement of LC-PUFAs across aquatic and terrestrial ecosystems, highlighting their transfer through food webs and the role of aquatic organisms as vectors of essential FAs across ecosystem boundaries. We also discuss how spatial variation in n-3 LC-PUFA availability influences ecological strategies, habitat selection, and adaptation of aquatic consumers to contrasting nutritional environments. Finally, the review highlights future opportunities in LC-PUFA research, including enzyme engineering, multi-omics-assisted breeding, functional genomics, alternative feed resources such as microalgae and insects, and integrated multitrophic aquaculture (IMTA), all of which hold significant potential for improving LC-PUFA production and supporting sustainable aquaculture development.

## 2. Bibliometric Analysis

### 2.1. Data Collection

In this study, we used Scopus as the primary source database because it covers peer-reviewed literature across the life sciences, engineering, and related disciplines. It provides comprehensive search options, complete author and institutional descriptions, and reliable citation parameters such as the CiteScore. This makes it particularly suitable for bibliometric studies. Its reliability and extensive indexing provide a robust foundation for mapping research trends at the intersection of LC-PUFA biosynthesis in aquatic animal studies.

To focus exclusively on the most relevant studies, the search was restricted to titles, abstracts, and keyword fields. The literature retrieval timeframe ranged from 1991 to 2025, encompassing 35 years of scholarly work. This range enables an in-depth evaluation of historical developments, scientific milestones, and emerging research directions concerning LC-PUFA biosynthesis in aquatic systems and aquatic species.

The search strategy was refined through multiple iterations to maximize recall (comprehensiveness) and precision (relevance). Several rounds of keyword testing and logical combination adjustments were performed before finalizing the search string: (“LC-PUFA biosynthesis” OR “LCPUFA” OR biosynth* OR “Omega-3 biosynthesis” OR “eicosapentaenoic acid biosynthesis” OR “docosahexaenoic acid synthesis” OR metabolism OR conversion OR elongat*) AND (fish OR teleost OR shellfish OR crustacean OR mollusc OR “aquatic animal”) AND (fads* OR “fatty acyl desaturase” OR elovl* OR *elovl2* OR *elovl5* OR “fatty acid elongase” OR desaturase OR elongase). This query returned 1083 records as of 30 October 2025. The first component of the search string captures various terminologies associated with the biosynthetic and metabolic processes of LC-PUFAs, the second targets aquatic taxa, such as fish and invertebrates, and the third focuses on key genes encoding biosynthetic enzymes, such as Fads and Elovl families, responsible for desaturation and elongation reactions. To further refine the dataset, certain terms, such as pathway, gene expression, and lipid metabolism, were tested but ultimately excluded, as their inclusion retrieved many peripheral studies not related to LC-PUFA biosynthesis mechanisms. Therefore, the finalized search string balances breadth and specificity, ensuring the collection of a sufficiently large yet focused dataset for robust bibliometric analyses.

Despite careful refinement, minor inconsistencies were inevitable in bibliometric data collection. Some relevant studies, particularly early, interdisciplinary, or non-English publications, may not have appeared because of variations in terminology. Meanwhile, a few unrelated articles may have been included unintentionally. These minor discrepancies are common in large-scale bibliometric analyses and are acceptable within the scope of this review.

### 2.2. Bibliometric Tools

High-quality bibliometric research relies on clear, accurate, and visually engaging data presentation. Therefore, the choice of appropriate analytical and visualization tools plays a key role in ensuring both academic rigor and interpretability of the results. In this study, VOSviewer (version 1.6.20) was used for network mapping and analysis, whereas Microsoft Excel, ChiPlot (https://www.chiplot.online/), and Flourish (https://flourish.studio/) were used to organize, analyze, and visualize the bibliometric data.

VOSviewer is well-established visualization software designed by Nees Jan van Eck and Ludo Waltman at the Center for Science and Technology Studies, Leiden University [29]. It was designed for the construction and exploration of bibliometric networks. The software can generate maps that display patterns such as keyword co-occurrence and co-authorship, institutional linkages, and journal co-citations. One of its strengths lies in handling large datasets efficiently while allowing users to adjust visualization parameters such as node size, label density, and link strength for clearer interpretation. Dynamic features, including scaling and grouping, further enable the detailed exploration of research structures and trends.

From a methodological perspective, VOSviewer can be used to construct a similarity matrix to measure the relationship between items, such as keywords, authors, or institutions, based on their co-occurrence frequency. The resemblance between items *a* and *b* is expressed as follows [30]:

(1) $$W_{\mathrm{ab}}=\frac{f_{\mathrm{ab}}}{u_{a}u_{b}}$$

where *W_ab_* represents the relatedness of items *a* and *b*; *f_ab_* is the number of co-occurrences between them; and *u_a_* and *u_b_* denote the total number of occurrences.

During visualization, the algorithm attributes spatial positions to items by minimizing the aggregate weighted value of the squared Euclidean pairwise distances among all items [29,30]

(2) $$F\left(x_{1},\;x_{2},\;\ldots ,x_{n}\right)=\sum_{a<b}W_{\mathrm{ab}}{\left\|x_{a}-x_{b}\right\|}^{2}$$

subject to the centering constraint

(3) $$\frac{2}{n(n-1)}\sum_{a<b}W_{\mathrm{ab}}\left\|x_{a}-x_{b}\right\|=1.$$

Here, *n* is the sum of all items, and *x_a_* is the position vector of item *a*. This ensured that items with strong similarity appeared near each other in the network, yielding an intuitive visual layout that highlighted thematic clusters and research linkages.

Network analysis provides insights into research dynamics by examining the co-occurrence patterns among keywords, authors, citations, and countries. Such analyses help to identify influential authors, institutions, and collaborative networks and highlight core themes and evolving directions within a research field. Network analyses were conducted for country collaborations, author collaborations, and overall keywords, as discussed in Section 2.8.

Microsoft Excel was used for data preprocessing and tabulation because of its flexibility, accessibility, and compatibility with other analytical tools. To enhance the graphical presentation of the bibliometric results, ChiPlot and Flourish were used to create high-quality interactive visualizations. These tools provide advanced customization options and design flexibility, overcome the visual limitations of standard spreadsheet graphics, and ensure clear, engaging, and publication-ready figures.

It should be noted that the temporal analysis was based on absolute publication counts and was not normalized against the overall growth of scientific output in broader related fields. Therefore, increases in publication numbers should be interpreted as growth in publication activity within the retrieved literature rather than as evidence of increasing relative specialization or research interest. Future bibliometric studies could incorporate normalized indicators, such as the Activity Index, to evaluate topic-specific growth relative to broader disciplinary publication trends.

### 2.3. Publication Analysis

#### 2.3.1. Annual Publication Trends

**Figure 1:**
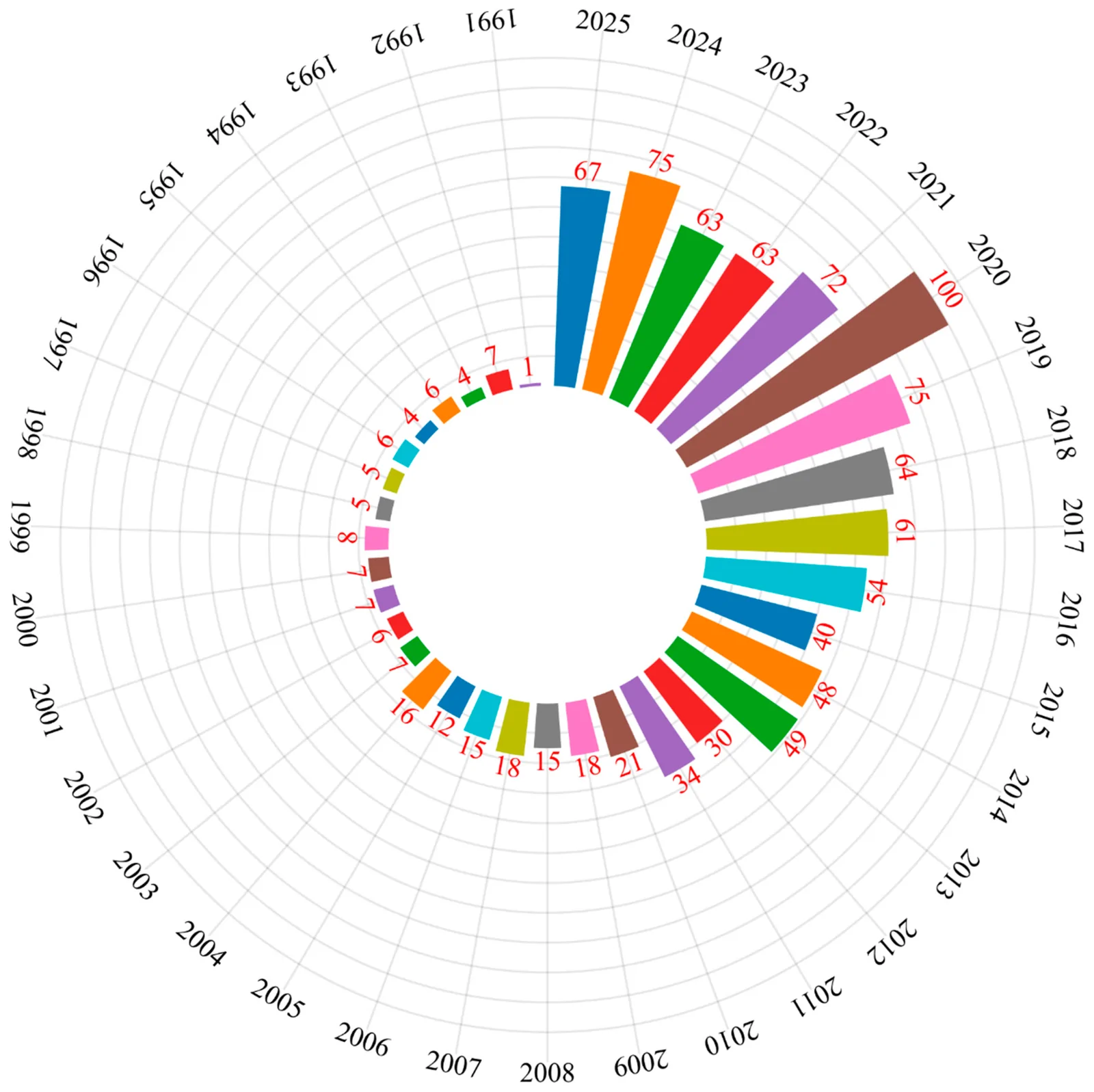
Annual publication counts from 1991 to 2025.

The annual publication output on LC-PUFA biosynthesis in aquatic animals from 1991 to 2025 was compiled and analyzed to identify long-term research trends. Bibliometric data were retrieved from the Scopus database (document search results; https://www.scopus.com). The results were visualized using an arc petal chart (Figure 1), which illustrates the changes in publication volume over the past 35 years.

Figure 1 shows clear temporal changes in publication output in this field. The period between 1991 and 2005 represents the formative stage, marked by a modest publication rate, ranging from one to 16 papers annually. This era was characterized by pioneering biochemical and enzymatic studies that focused on the identification and characterization of desaturases and elongation in key aquatic species. Progress has been constrained by the limited availability of molecular tools and a strong emphasis on descriptive physiology rather than molecular mechanisms.

A transitional growth phase emerged from 2006 to 2015, with output steadily increasing from 21 in 2008 to 54 in 2015. This period coincided with the advent of genome sequencing in major aquaculture species, such as salmon and tilapia, and increased the recognition of the roles of LC-PUFAs in immunity, development, and environmental adaptation. The integration of transcriptomics and functional genomics marked a shift toward mechanistic and applied research, setting the stage for accelerated progress.

The largest increase in publication output occurred after 2015, reaching 100 publications in 2020. This period coincided with several important developments in the field, including a decline in wild fish stocks, rising demand for sustainable ω-3 sources, advances in CRISPR-Cas9 gene editing, and policy-driven strategies in aquaculture nutrition. The post-peak stabilization of 60–75 papers annually signals a maturing field with sustained momentum, creating ample opportunities for deeper mechanistic insights, novel biotechnological innovations, and cross-species comparative studies. Overall, the temporal pattern demonstrates a substantial increase in LC-PUFA-related publication output, particularly after 2015, together with diversification toward mechanistic, genomic, and applied research.

Table 1 summarizes the distribution of document types. Original research articles dominated the dataset (1005 papers, 92.8%), highlighting the strong experimental and data-driven nature of this field. Review papers (46, 4.25%) played an important role in synthesizing progress and shaping future directions. Book chapters (nine; 0.83%), conference papers (five; 0.46%), and other publication types contributed marginally, indicating that research dissemination was primarily concentrated in peer-reviewed journals. English was the predominant language, accounting for more than 98.5% of all publications and reflecting the field’s high level of international engagement.

#### 2.3.2. Country-Wise Publications

After ranking the publication output of the 20 countries from highest to lowest, we found that China, the United Kingdom, Spain, the United States, and Australia were the leading contributors, with 360, 176, 153, 146, and 73 publications, respectively. This distribution highlights the prominent research in this field.

**Figure 2:**
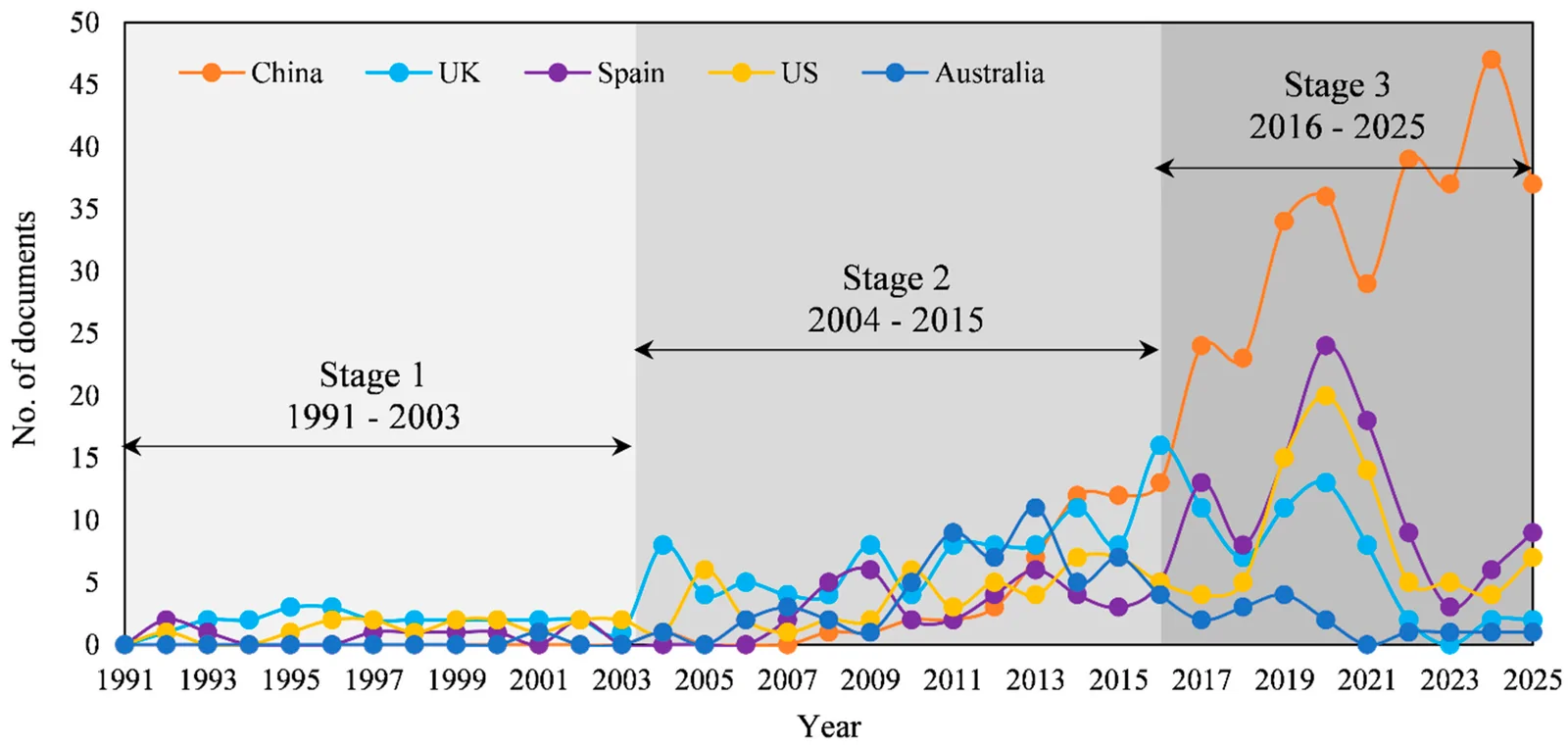
Annual publication output of the top five countries.

Figure 2 illustrates the annual publication output of the top five countries contributing to LC-PUFA biosynthesis in aquatic animals, divided into three developmental stages. Stage 1 (1991–2003) shows sparse and evenly distributed activity, with all countries producing fewer than five papers annually. This reflects limited but foundational research in a few pioneering laboratories. Stage 2 (2004–2015) marks the emergence of leadership, as China initiated a steep upward trajectory in 2008, surpassing others by 2012, and reaching approximately 15 papers/year by 2015, driven by expanding aquaculture research infrastructure and national funding priorities. The UK, Spain, and the US display moderate fluctuating growth (5–10 papers/year), whereas Australia remains lower but consistent.

Stage 3 (2016–2025) highlights the clear dominance of China, with outputs surging to over 40 papers in 2022 before stabilizing at approximately 35–40 annually, accounting for most global growth. The UK and Spain followed with secondary peaks of approximately 15–20 papers/year. Meanwhile, the US and Australia plateaued or declined slightly after 2020, suggesting saturation in traditional research models. The overall trend underscores China’s pivotal role in advancing the field through large-scale genomic, nutritional, and biotechnological studies, while established Western contributors maintain a steady but less expansive output. This growing disparity suggests the need for stronger international collaboration to combine the research volume of China with the specialized expertise of the UK, Spain, and others.

The strong contribution of these countries may reflect a combination of their established aquaculture and fisheries sectors, long-standing research capacity in aquatic nutrition and lipid metabolism, and sustained investment in marine and aquaculture sciences. This association is particularly evident for China, where the rapid expansion of aquaculture has been accompanied by substantial research activity in fish nutrition, genomics, and sustainable feed development. Spain and the United Kingdom also have well-established marine and aquaculture research programs, while the United States and Australia contribute substantial expertise in aquatic biology, nutrition, and fisheries research. Thus, the geographical distribution of publications likely reflects not only the scale of aquaculture production but also differences in national research infrastructure, funding priorities, and established expertise in aquatic sciences [31].

### 2.4. Citation Analysis

**Figure 3:**
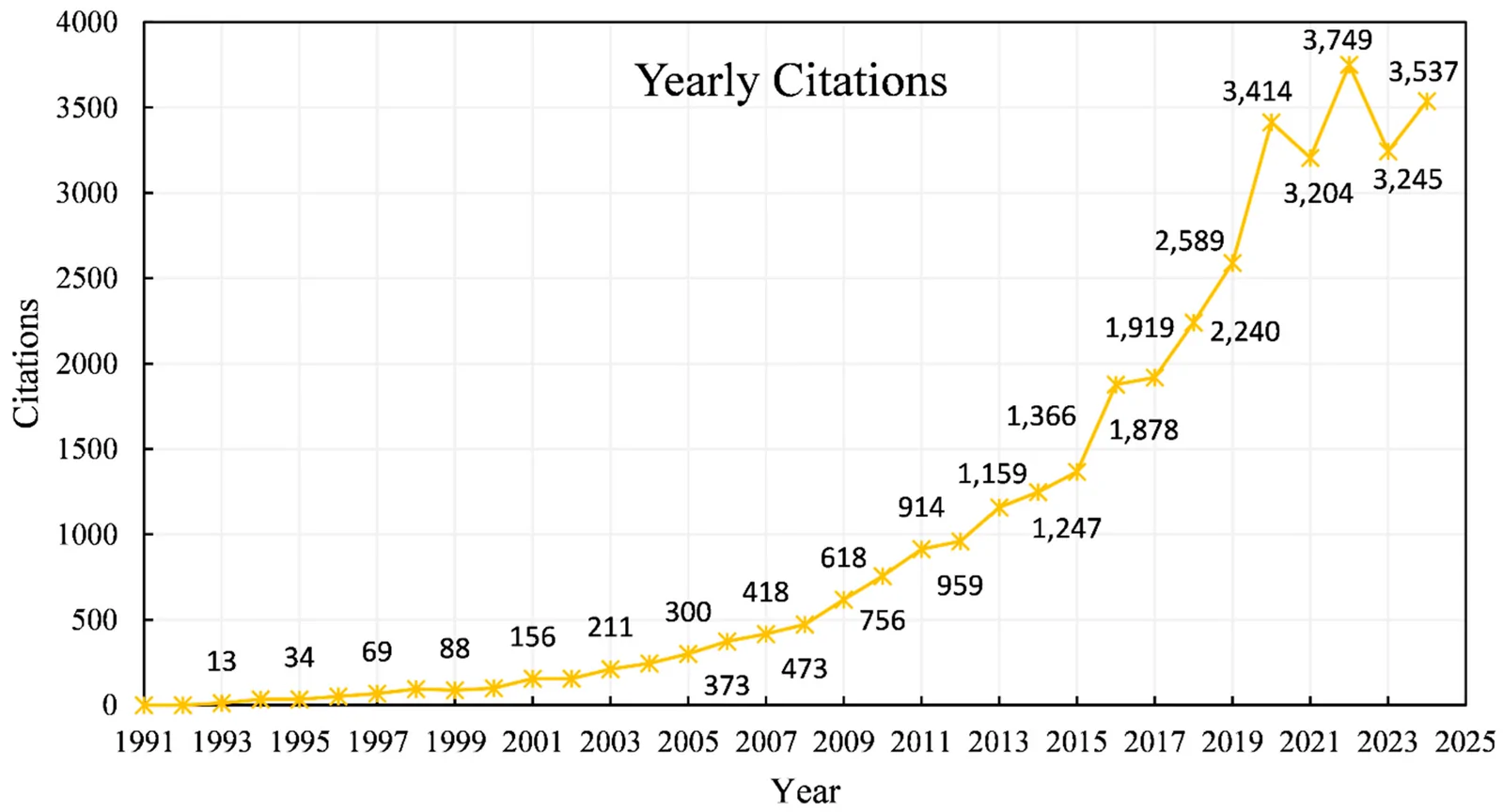
Annual citation trends for publications (Scopus 1991–2024).

Citation counts are widely regarded as indicators of the influence and scientific importance of a publication. Analyzing the most frequently cited works, their methods, findings, and innovations can provide meaningful perspectives and directions for future research. Figure 3 shows the annual citation trends for LC-PUFA biosynthesis in aquatic animals (1991–2024). Annual citations remained negligible (<100/year) during the early phase (1991–2000) and then increased steadily from 2001, reaching approximately 1000 per year by 2015 as genomic tools and ω-3 nutrition gained prominence in aquaculture. A sharp surge after 2016, peaking at 3749 citations in 2022 and stabilizing near 3500 annually, aligns with the rapid increase in publication volume (Figure 1) and the shift toward high-impact applications, such as gene editing, synthetic biology, and sustainable feed development. The slight decrease between 2023 and 2024 likely reflects citation delays, with the recovery in 2025 indicating ongoing vitality. This trend confirms that LC-PUFA biosynthesis has become a high-impact research area, with cumulative citations surpassing 30,000.

The citation frequency of an article depends on multiple factors; however, it remains a widely recognized indicator of scientific influence. Table 2 lists the ten most cited documents on LC-PUFA biosynthesis in aquatic animals, which have accumulated over 2500 citations and established the intellectual groundwork of the field.

Among the earliest and most influential studies, Henderson (1996) and Hastings et al. (2001) provided foundational insights [14,32]. Henderson (1996, 289 citations) demonstrated that freshwater fish can synthesize saturated and monounsaturated FAs de novo, but lack the full enzymatic suite to produce PUFAs independently, necessitating dietary intake of these essential lipids [32]. He further emphasized that the dietary n-3:n-6 ratio critically modulates eicosanoid synthesis, shaping fish physiology and nutritional status. Building on the biochemical foundation, Hastings et al. (2001, 308 citations) identified a vertebrate desaturase in zebrafish exhibiting both Δ5 and Δ6 activities, an enzymatic duality absent in mammals [14]. When expressed in *S. cerevisiae*, this bifunctional enzyme converts C18 and C20 precursors into arachidonic and eicosapentaenoic acids, representing a key breakthrough in the understanding of vertebrate PUFA biosynthesis. The most-cited review article by Castro et al. (2016, 364 citations) provided an evolutionary synthesis of *Fads* and *Elovl* gene repertoires across chordates [13]. Their work highlighted how genome duplication and functional diversification shaped the LC-PUFA biosynthetic capacity among vertebrates, offering a genetic and phylogenetic framework that continues to guide modern research.

Geographically, the UK has six of the top-cited papers, followed by China, Spain, Portugal, and France, mirroring their strong research output. All the highly cited works appear in prominent journals such as PNAS and Progress in Lipid Research, with impact factors ranging from 1.5 to 14.9, showing that both scientific quality and publication venue influence citation success. The mix of original studies and reviews highlights how discovery and synthesis drive progress in functional genomics, nutrition, and aquaculture biotechnology.

### 2.5. Leading Institutions by Country

**Figure 4:**
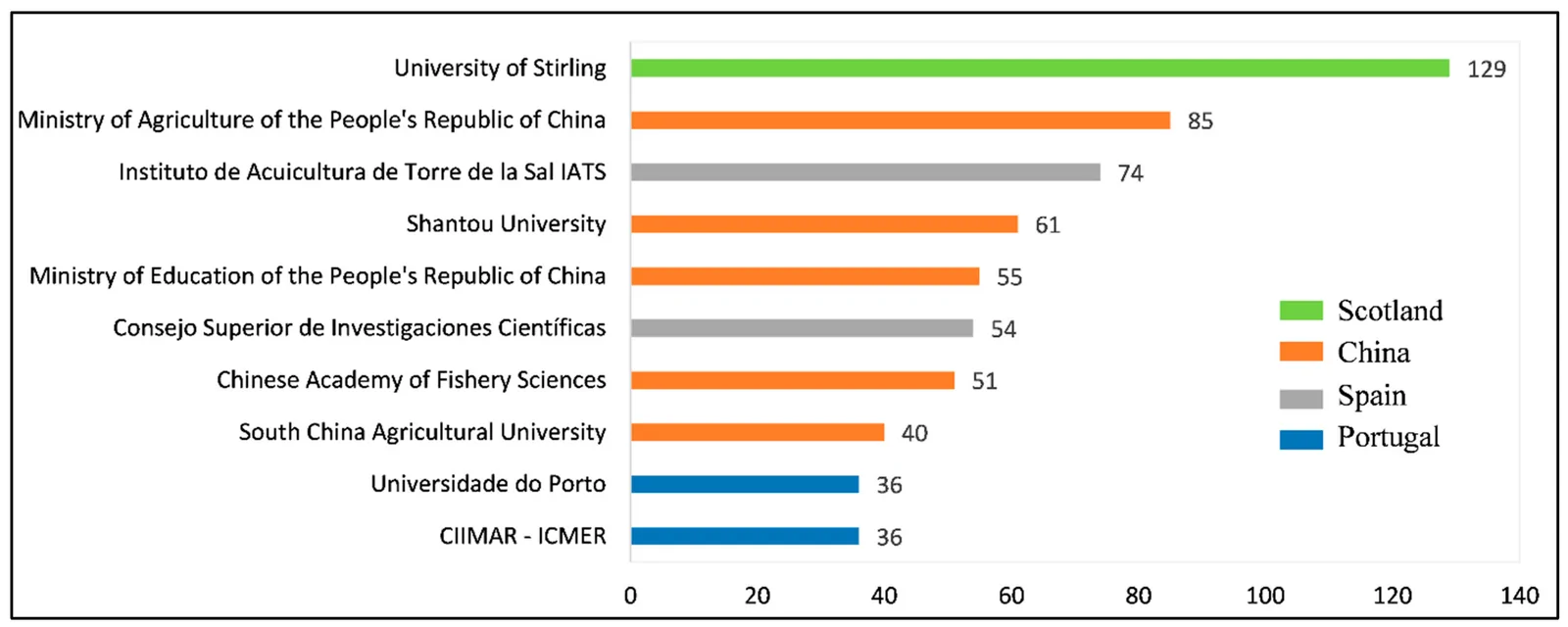
The 10 most productive institutions in LC-PUFA research during 1991–2025.

Figure 4 highlights the top contributing institutions in LC-PUFA biosynthesis in aquatic animals, with publication outputs color-coded by country. The University of Stirling (Scotland) leads with 129 documents, reflecting its long-standing excellence in aquaculture nutrition and lipid metabolism research. China dominates the ranking with five institutions, including the Ministry of Agriculture (85), Shantou University (61), the Ministry of Education (55), the Chinese Academy of Fishery Sciences (51), and South China Agricultural University (40), collectively underscoring the state-driven, multi-institutional push in applied aquatic biotechnology in China.

Spain has two major institutions, Instituto de Acuicultura de Torre de la Sal (IATS) (74) and Consejo Superior de Investigaciones Científicas (CSIC) (54), which demonstrate strong public sector leadership in marine lipidomics. Portugal is represented by the Universidade do Porto (36) and CIIMAR-ICMER (36), both of which emphasize interdisciplinary marine and environmental approaches. This distribution mirrors national research priorities: Scotland and Europe focus on fundamental and translational science, whereas China leverages centralized funding and scale to drive high-volume, solution-oriented output. The prominence of government-affiliated bodies, for example, Chinese ministries and CSIC, signals strategic policy alignment with the global demand for sustainable ω-3 production in aquaculture.

### 2.6. Most Productive and Influential Authors

Table 3 lists the top 10 most productive authors contributing to LC-PUFA biosynthesis research in aquatic animals, combining publication and citation metrics to highlight productivity and impact. Defined as the number of papers (*h*) that have each been cited at least *h* times, the h-index is a robust indicator of sustained scholarly influence [40]. Tocher (University of Stirling, UK) led with 121 publications and 9145 citations (h-index, 76), establishing dominance through decades of foundational work. Monroig, Ó. (CSIC, Spain) (91 documents, 4783 citations, h-index 53), reflecting sustained leadership in fish nutrition, lipid biochemistry, and molecular biology.

Chinese researchers occupy four spots, with Li, Y., and Wang, S. (South China Agricultural University; Shantou University) combining high output (41–41 documents) with high citation efficiency (ACPD 37–35). Mai (Ocean University of China) stands out for its exceptional impact per paper (ACPD 36, h-index 83, TCO 27,144), despite fewer documents (20), highlighting the strategic focus on high-visibility journals.

Dick, J.R. (University of Stirling, UK) achieved the highest ACPD (108) and 2817 citations, underscoring the translational influence in complex lipid analysis. The range of h-indices (24–83) and diverse institutional affiliations reflect a well-established and globally interconnected research community. Early pioneers such as Tocher and Navarro built the foundation. Meanwhile, emerging contributors such as Li and Mai drove new frontiers in FA metabolism and aquaculture biotechnology. The synergy between experience and innovation continues to propel this field.

### 2.7. Leading Journals

Identifying leading journals enables researchers to follow key studies, trace knowledge dissemination, and target the most influential outlets for their work. Table 4 lists the top 15 most productive journals on LC-PUFA biosynthesis in aquatic animals, with *Aquaculture* leading with 107 documents (9.88%) and an h-index of 40, underscoring its role as the primary venue for applied aquaculture nutrition studies. *Comparative Biochemistry and Physiology-B* (39 documents, IF 1.8) and *PLoS One* (29 documents, IF 2.6) followed, highlighting the reliance of this field on high-volume multidisciplinary platforms. Specialized journals such as *Lipids* (27 documents, IF 1.6) and *Fish Physiology and Biochemistry* (22 documents, IF 2.5) cater to biochemical and physiological inquiries. Meanwhile, *Aquaculture Nutrition* (19 documents, IF 3.9) and *International Journal of Molecular Sciences* (16 documents, IF 4.9) indicate an emphasis on molecular mechanisms.

When selecting a target journal, researchers should evaluate the impact factor (IF) for prestige, the h-index for sustained influence, the acceptance rate for feasibility, and scope alignment to ensure a topical fit. Ultra-high IF journals, such as *Progress in Lipid Research* (IF 14.9) and *PNAS* (IF 9.1), have published landmark reviews and articles in this domain [13] yet contribute minimally in volume owing to strict selectivity, low acceptance rates (<10%), and a broader editorial scope not exclusively tailored to aquatic LC-PUFA research.

**Figure 5:**
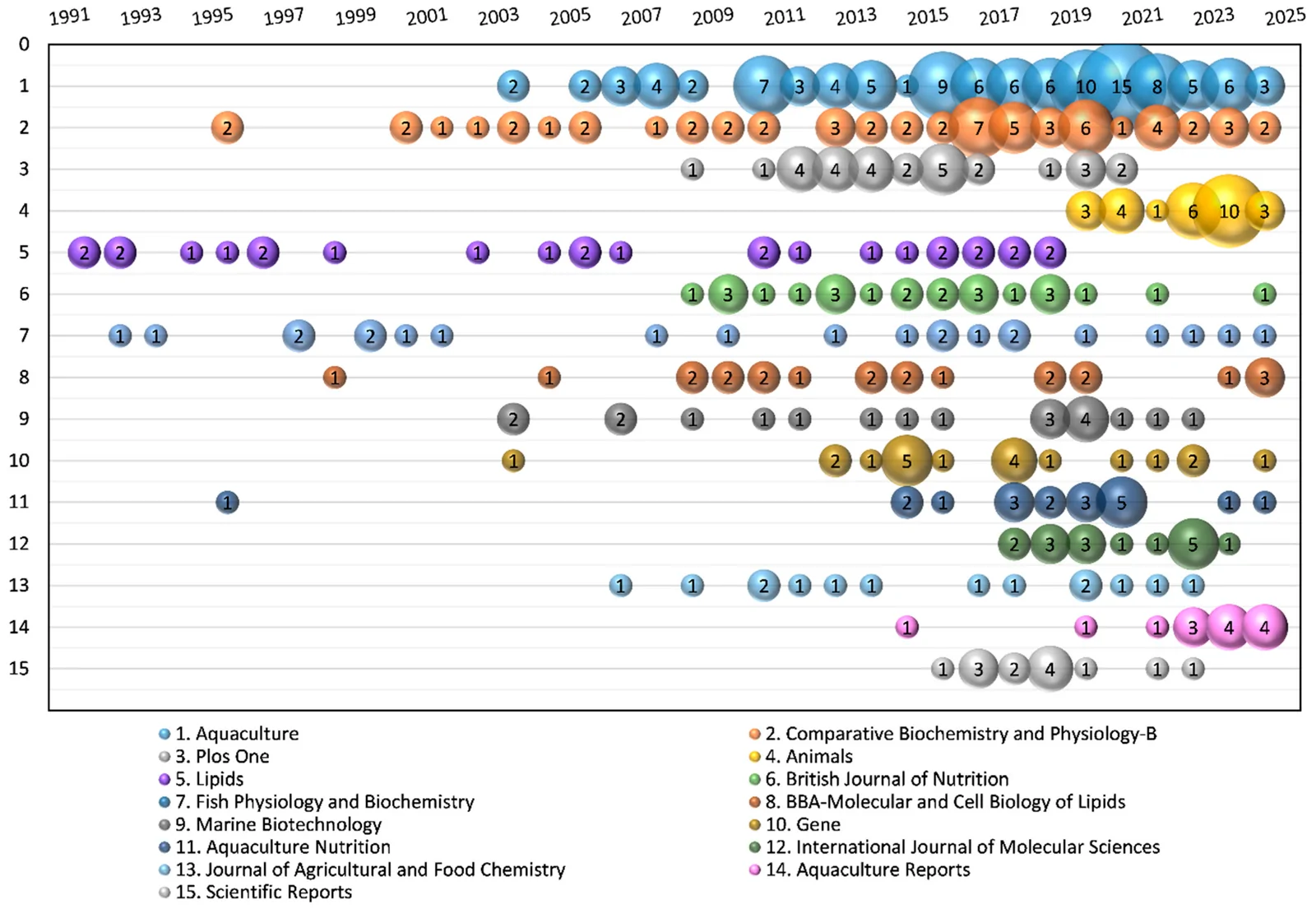
Bubble chart of the top 15 productive journals by year.

Figure 5 complements this analysis using a temporal bubble chart that reveals three publication phases. The early period (1991–2005) showed small, scattered contributions from *Aquaculture* (blue) and *Lipids* (purple), consistent with foundational work. The expansion phase (2006–2015) featured rapid growth in *PLoS One* (gray) and *British Journal of Nutrition* (green), driven by genomic and open-access trends. Post-2015, *Animals* (gray) and *Aquaculture Reports* (blue) emerged prominently, along with *Aquaculture* (lavender) and *Comparative Biochemistry and Physiology-B* (orange), with bubble sizes reaching 10–15 documents/year. This reflects the shift in this field toward high-throughput, multidisciplinary publishing. *Comparative Biochemistry and Physiology-B* maintained a consistent mid-level output across decades, while newer entrants, such as the *International Journal of Molecular Sciences*, gained momentum after 2017.

These insights indicate a strategic publication landscape. Core field-specific journals ensure depth and continuity. Meanwhile, open-access multidisciplinary platforms accelerate dissemination and citation potential. This dual structure supports specialized advancements and global accessibility, guiding authors toward venues that optimize their impact within realistic submission success rates.

### 2.8. Network Analysis

#### 2.8.1. Co-Authorship Network

Co-authorship analysis provides key insights into the collaborative study design and structure. This highlights how researchers connect and share knowledge and identifies leading authors and active research clusters. This reveals the collaborative patterns that shape the emergence of the field.

**Figure 6:**
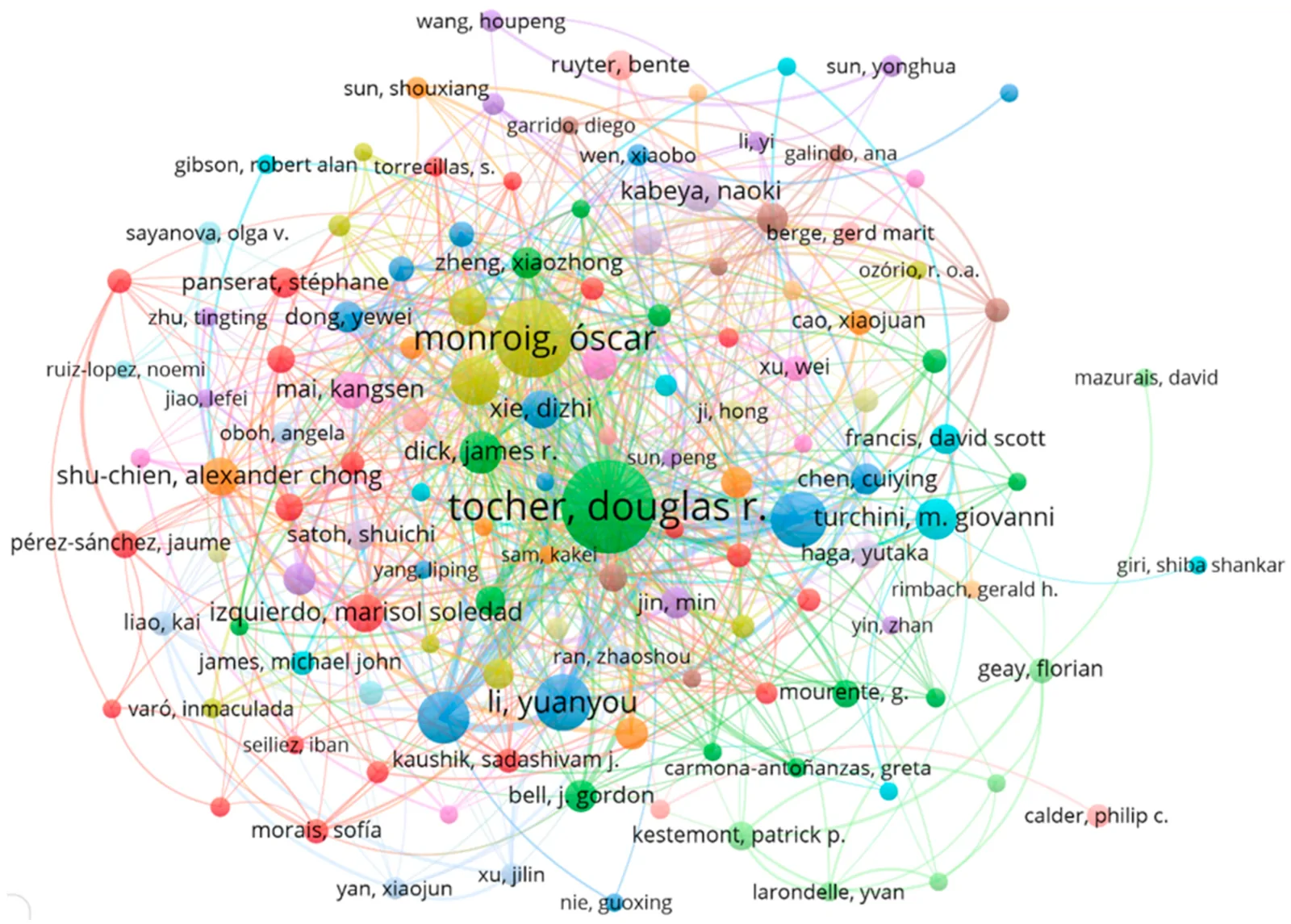
Co-authorship network map.

Figure 6 illustrates the collaborative authorship network generated using VOSviewer, which visualizes collaborative relationships among authors. A threshold value of at least five publications for each author was set, resulting in 163 qualifying authors out of a total of 4342. For clarity of visualization, the top 130, demonstrating the greatest total link strength, were included. The analysis used “Documents” as the weight parameter, with attraction and repulsion values of 8 and −8, respectively. In the network map, each node represents an author. Meanwhile, the node size indicates the extent of collaboration. The larger the node, the stronger the cooperative activity. Different colors distinguish clusters of closely connected authors, signifying distinct research groups.

The visualization showed a clear clustering structure, indicating the existence of multiple active and stable collaborative groups in the research field. A total of 16 clusters were identified. Among these, Tocher Douglas exhibited the highest total link strength (407), demonstrating strong research partnerships. He is followed by Monroig Óscar (372) and Li Yuanyou (201). Other prominent contributors included Wang Shuqi, Cuihong You, Navarro Juan Carlos, and Kabeya Naoki, each with link strengths exceeding 100. These highly connected authors act as central hubs that facilitate cooperation and knowledge exchange within the community. Conversely, a few isolated or weakly connected nodes indicated independent researchers, likely because of smaller research groups or limited opportunities for cross-institutional collaboration.

#### 2.8.2. Country Collaboration Network

**Figure 7:**
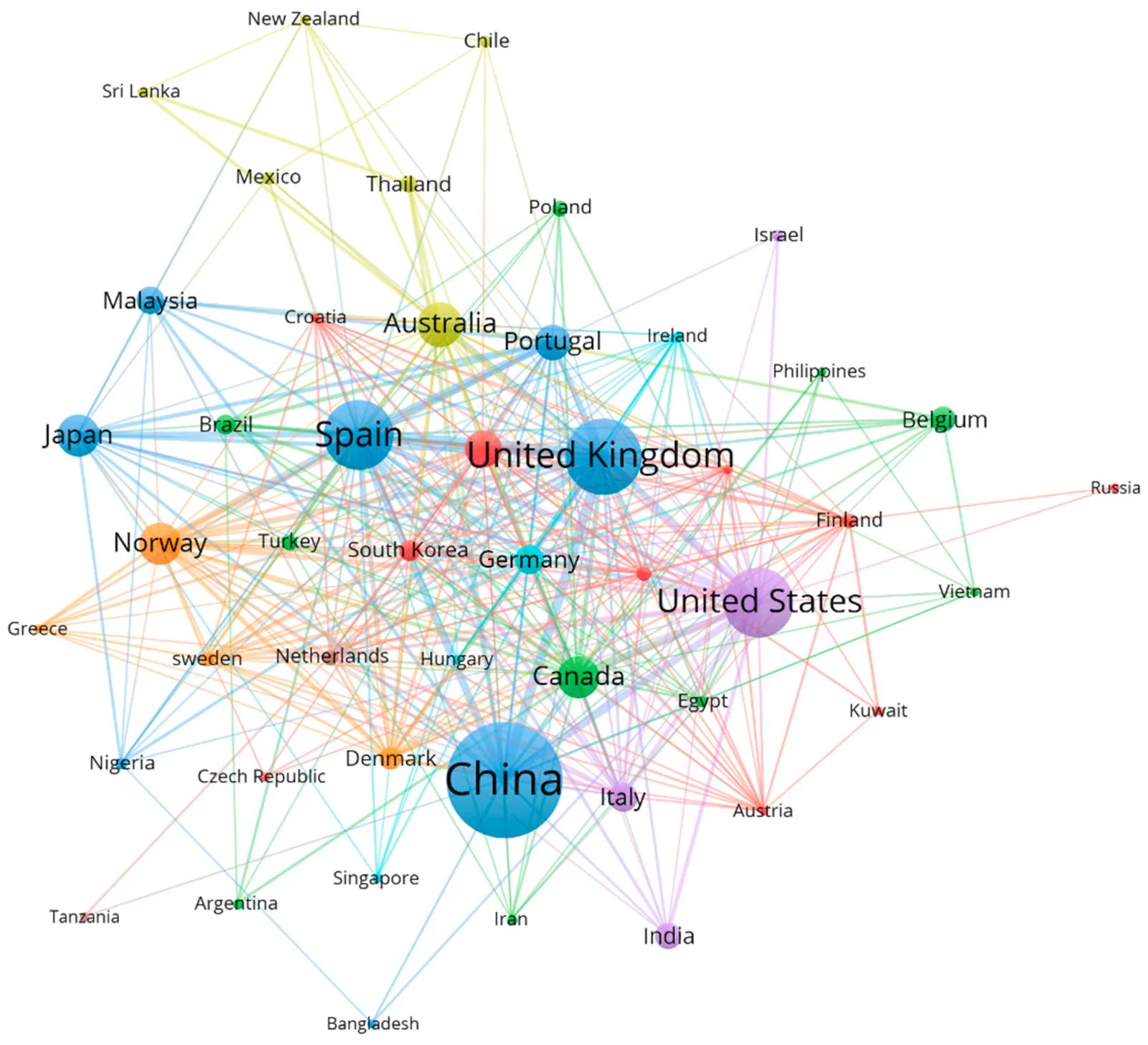
Country collaboration network.

Figure 7 illustrates the international collaboration network for LC-PUFA biosynthesis in aquatic animals. A total of 68 countries were identified. By setting a minimum publication threshold of three documents, 49 countries met the criteria for inclusion in the analysis. Node size reflects publication volume. Meanwhile, link thickness indicates co-authorship strength, showing a densely interconnected core dominated by China, the USA, the UK, Spain, Norway, and Australia. China occupies the central hub, forming robust partnerships with the USA, the UK, Australia, and emerging aquaculture nations, such as Vietnam and Thailand, reflecting its dual role as a high-output leader and collaborative bridge. The USA and the UK serve as secondary hubs, linking European (Spain, Norway, Germany) and Asia-Pacific (Japan, South Korea) clusters and facilitating transcontinental knowledge flow.

Peripheral nodes, such as Egypt, Sri Lanka, and Tanzania, connect primarily through single ties to core countries, suggesting nascent but growing engagement. This hierarchical structure highlights a collaborative landscape driven by shared aquaculture interests, technological complementarity, and funding synergies, such as EU–China joint programs and bilateral UK–Australia initiatives. The high density of this network within the core highlights mature multilateral exchange. Meanwhile, sparse outer links signal opportunities for capacity-building partnerships to integrate underrepresented regions into global research efforts on LC-PUFA biosynthesis.

#### 2.8.3. Keyword Co-Occurrence Density Map

**Figure 8:**
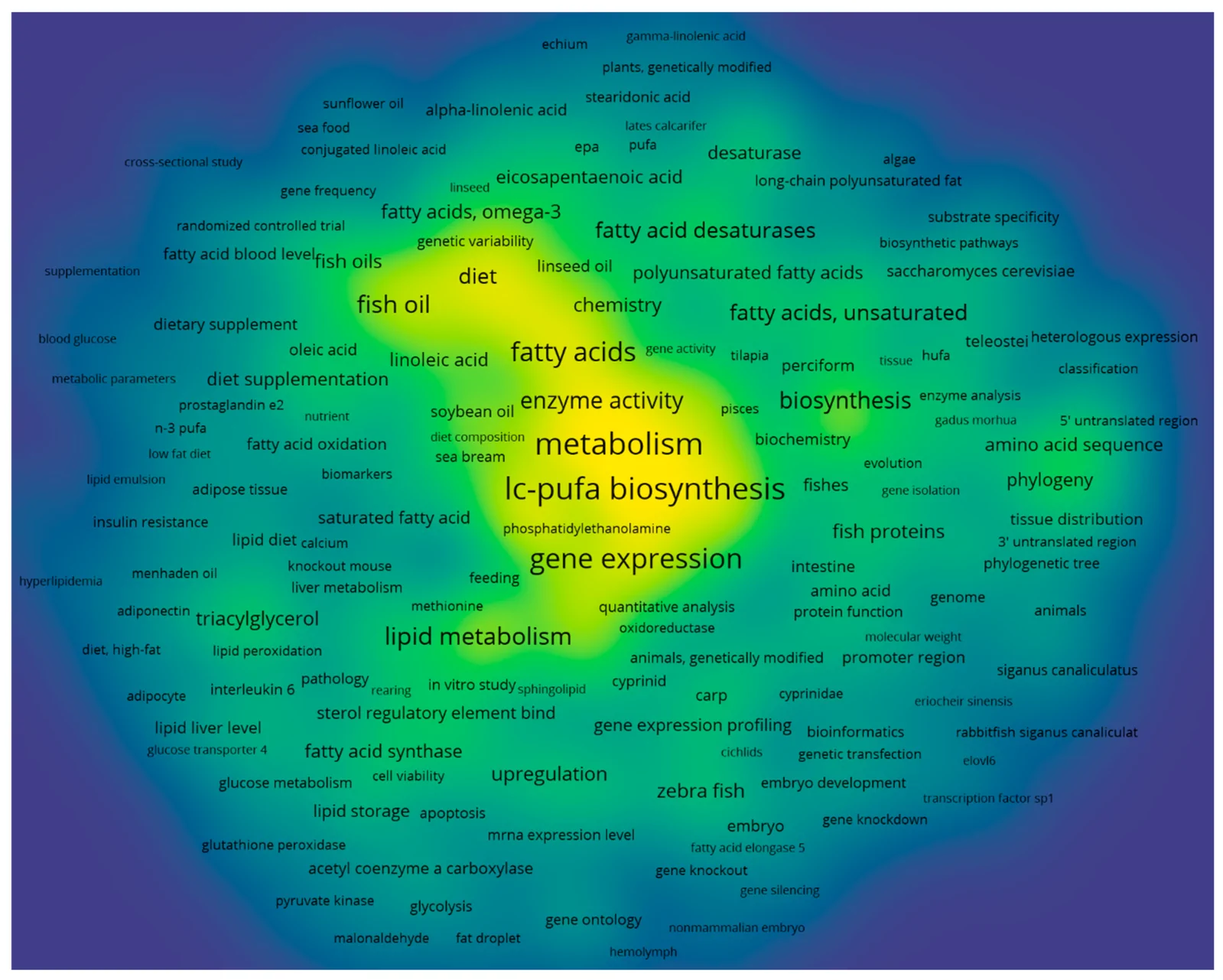
Keyword co-occurrence density map.

Figure 8 shows a density map of keyword co-occurrence generated from the full bibliographic dataset (1991–2025). All the keywords assigned by authors and indexing services were analyzed subject to a minimum co-occurrence threshold of 5. Out of the 9867 extracted keywords, 1287 met this criterion. The resulting visualization showed a highly structured research landscape centered on the core biochemical and molecular themes of LC-PUFA biosynthesis. Central high-frequency terms, including “lc-pufa biosynthesis,” “metabolism,” “gene expression,” “enzyme activity,” “fatty acids,” “fatty acid desaturases,” “elongase,” and “biosynthesis”, highlight that mechanistic elucidation of metabolic pathways remains the foundation of the field. Surrounding clusters point to major model organisms (zebrafish, tilapia, carp, marine fish) and common experimental techniques such as heterologous expression, gene knockout, and promoter region analysis. A strong nutrition-related cluster appeared around fish oil, linseed oil, soybean oil, fatty acid oxidation, and dietary supplementation, underscoring long-standing efforts to develop alternative lipid sources and to understand the dietary regulation of LC-PUFA metabolism.

Several emerging research focus areas are visible in moderately dense (green-yellow) regions. Terms such as “phosphatidylethanolamine,” “phospholipid,” “lipid metabolism,” “lipid peroxidation,” and “triacylglycerol” indicate an emphasis on holistic lipidomic profiling rather than gene-level characterization alone. Keywords including “salinity,” “intestine,” “tissue distribution,” and “pathology” indicate research attention to the environmental and physiological modulation of LC-PUFA biosynthesis across species. The prominence of “gene expression profiling,” “upregulation,” “gene ontology,” and “mRNA expression level” suggests continued progression toward integrative molecular tools that link transcriptomic responses with metabolic function.

The density map depicts a mature and cohesive research domain anchored in biochemical mechanisms but is increasingly enriched by lipidomics, dietary interventions, and environmental physiology. These clusters highlight potential directions for further research, including multi-omics integration, refined nutritional strategies, and a deeper understanding of the environmental modulation of LC-PUFA biosynthesis in aquatic species.

## 3. Thematic Review

Building on bibliometric analysis, which maps the temporal development, structure, and collaborative patterns of LC-PUFA research, this section shifts the focus from where and how often studies have been published to what they reveal. This thematic review provides key biological, molecular, and regulatory insights underlying LC-PUFA biosynthesis in aquatic animals. By integrating findings across species, experimental systems, and regulatory levels, this review provides a coherent narrative of the current insights into LC-PUFA acquisition, biosynthesis, and ecological roles in aquatic animals and provides a basis for identifying knowledge gaps and guiding future research.

### 3.1. Primary Producer and Trophic Transfer of LC-PUFA

Unicellular eukaryotic microalgae are the predominant natural producers of LC-PUFAs. These organisms represent a sustainable, plant-based, and environmentally friendly source for the biotechnological production of LC-PUFAs, offering a viable alternative to fish oil [41]. In aquatic ecosystems, microalgae are major primary producers of LC-PUFAs, including EPA and DHA, and constitute an important source of these FAs at the base of aquatic food webs [20,42]. Other microorganisms, including certain heterotrophic protists and bacteria, can also contribute to LC-PUFA production and availability at lower trophic levels. Marine microorganisms produce PUFAs through diverse biosynthetic pathways, including aerobic desaturase/elongase and anaerobic polyketide synthase (PKS) mechanisms, which have been extensively reviewed [43,44].

The traditional view that microalgal LC-PUFAs are simply transferred unchanged along food chains is incomplete because consumers can selectively retain and metabolically modify dietary FAs during trophic transfer [45]. These processes occur within complex and interconnected aquatic food webs. Mehner et al. [46], for example, illustrated the linkages among microbial, pelagic, benthic, and terrestrial resources in lake food webs. Aquatic and terrestrial ecosystems are further connected through reciprocal fluxes of biomass and FAs. Makhutova et al. [47] provided quantitative evidence of such cross-ecosystem linkages in a shaded river, demonstrating substantial differences between terrestrial invertebrate inputs to the aquatic ecosystem and the export of emergent aquatic insects to the terrestrial environment. Importantly, these resource pathways differed in their PUFA composition, with terrestrial invertebrates providing relatively lower n-3 and higher n-6 PUFA fluxes than aquatic invertebrates. Thus, cross-ecosystem subsidies can influence not only the quantity of resources available to consumers but also their FA composition and nutritional quality.

The dietary transfer of preformed LC-PUFAs through aquatic food webs is particularly important because LC-PUFA availability and endogenous biosynthetic capacity differ between marine and freshwater fishes. Marine food webs generally provide preformed EPA and DHA, and many marine fishes consequently have limited capacity to biosynthesize these LC-PUFAs from C18 PUFA precursors. In contrast, LC-PUFA availability in freshwater food webs can be more variable, making both dietary composition and endogenous biosynthetic capacity particularly important. For example, Makhutova et al. [48] demonstrated that the EPA and DHA contents of Baikal grayling (*Thymallus baicalensis*) varied with dietary sources: fish relying more strongly on terrestrial invertebrates had lower EPA and DHA contents in muscle and adipose tissues than those relying predominantly on aquatic food resources. These differences illustrate how variation in dietary LC-PUFA supply can be reflected in the FA composition of freshwater consumers.

Beyond direct dietary transfer to fish, LC-PUFAs can also be selectively retained and modified as they pass through intermediate consumers in aquatic food webs. Consumers at higher trophic levels, including invertebrates and fish, can modify and further enrich the PUFA composition inherited from primary producers, a process referred to as trophic upgrading [33]. Essential PUFAs can be transferred through aquatic food webs more efficiently than bulk organic carbon, with EPA and DHA becoming selectively retained in consumer biomass [49,50]. Such selective retention, together with the preferential catabolism of non-essential compounds, can enhance the nutritional quality of organisms for subsequent trophic levels.

Heterotrophic protists are particularly important intermediates in this trophic transfer because they connect microbial production with larger consumers. Within the microbial loop, they channel energy and FAs from bacteria and picophytoplankton toward larger protists and small metazoans [51]. These organisms ingest a broad range of particles, including bacteria, cyanobacteria, and picophytoplankton, thereby contributing to nutrient recycling and secondary production in both marine and freshwater systems [52]. The PUFA composition of heterotrophic protists is influenced by interacting factors including habitat type, feeding strategy, and environmental temperature. Diet exerts a major influence on their FA profiles through the acquisition and selective retention of dietary PUFAs, whereas temperature can alter membrane FA composition through homeoviscous adaptation. In response to decreasing temperature, organisms commonly increase FA unsaturation to maintain membrane fluidity and physiological function [53,54]. Both marine and freshwater algivorous heterotrophic protists generally contain higher concentrations of n-3 PUFAs than bacterivorous species, largely because these FAs are accumulated directly from algal prey [55].

Despite evidence that different protist species can develop distinct PUFA profiles even when feeding on the same algal diet, the metabolic mechanisms underlying these differences remain incompletely understood [56]. This uncertainty limits predictions of how nutritional quality will be modified during trophic transfer under changing environmental conditions. The transfer and selective retention of n-3 PUFAs through successive trophic levels can improve the nutritional value of consumers for their predators [57]. Environmental changes, including shifts in temperature, nutrient availability, and light conditions, can alter the production and availability of n-3 PUFAs at the base of aquatic food webs. However, changes at higher trophic levels are not necessarily proportional to those occurring in primary producers because ecological processes such as selective herbivory, retention, and trophic upgrading can modify their transfer [58]. Consequently, the effects of climate-driven environmental changes on n-3 PUFA transfer through aquatic food webs are context-dependent and may ultimately influence their availability to higher trophic levels, with potential consequences for consumer growth, reproduction, and fitness [57,58].

### 3.2. Endogenous LC-PUFA Biosynthesis and Evolution Across Aquatic Animals

Although LC-PUFA availability in aquatic systems is largely influenced by primary production and food-web transfer, the ability of animals to produce these molecules through internal metabolic pathways represents an important evolutionary adaptation. This section therefore examines endogenous LC-PUFA biosynthesis in aquatic animals, focusing on how gene evolution, pathway diversification, and environmental selection pressures have generated species-specific differences in biosynthetic capacity. The capacity for endogenous LC-PUFA biosynthesis occurs across diverse vertebrate and invertebrate aquatic taxa, although comparative genomic and functional analyses reveal substantial lineage-specific variation in the underlying pathways and biosynthetic efficiencies [17,21]. Different species possess distinct combinations of Fads and Elovl enzymes, resulting in substantial variation in their capacity to synthesize major LC-PUFAs, including ARA, EPA, and DHA [59]. Consequently, endogenous LC-PUFA biosynthesis should be viewed as an adaptive evolutionary process rather than a universal metabolic pathway [60].

The evolutionary history of LC-PUFA biosynthesis reflects repeated episodes of gene duplication, functional divergence, and lineage-specific gene loss [21]. Early aquatic vertebrates likely possessed a relatively simple desaturation and elongation system [61]. Over evolutionary time, duplication events and subsequent functional diversification of *Fads* and *Elovl* genes contributed to the expansion and diversification of the LC-PUFA biosynthetic pathway [13]. Enzymes can evolve broader substrate recognition through promiscuous activities, while subsequent selection can promote greater catalytic specialization [62]. In some aquatic species, the loss or reduced activity of desaturase genes can limit endogenous LC-PUFA synthesis and increase reliance on dietary FAs [63]. Fatty acid metabolic capacity can vary substantially among species because of differences in their metabolic genes and enzymes [60].

Methyl-end (ωx) desaturases represent an important component of PUFA biosynthesis in several invertebrate lineages. Unlike conventional front-end desaturases, ωx desaturases introduce double bonds toward the methyl end of FAs. Although methyl-end desaturase activities are well established in plants and other organisms, their occurrence in animals appears to have arisen independently in several invertebrate lineages. These enzymes can enable the conversion of monounsaturated FAs into polyunsaturated FAs; for example, Δ12 and Δ15 desaturase activities contribute to the formation of LIN and ALA, respectively. Consequently, although de novo PUFA biosynthetic capacity is not a general characteristic of animals, it has been demonstrated in certain invertebrate lineages possessing functional methyl-end desaturases (e.g., molluscs, cnidarians, nematodes, rotifers, and crustaceans) [59]. The desaturase repertoires and functional diversity across various aquatic taxa are detailed in Table 5. Their occurrence demonstrates that the evolutionary origin of PUFA biosynthesis is more diverse than previously recognized [17]. Although complete de novo synthesis remains uncommon among vertebrates, these enzymes provide several invertebrate groups with an additional mechanism for maintaining membrane lipid homeostasis under conditions of limited dietary PUFA availability [21].

Following the production or dietary acquisition of C18 polyunsaturated FAs, further LC-PUFA synthesis depends largely on front-end desaturases [17,75]. These enzymes determine both the efficiency and flexibility of downstream metabolic pathways. Among them, Δ6 desaturase is one of the most ancient and widely distributed desaturases. It performs the first major desaturation step in both the n-3 and n-6 pathways [76]. Nevertheless, several aquatic species exhibit alternative Δ8 pathways [59]. In these species, elongation precedes desaturation, allowing eicosadienoic acid (20:2n-6) and eicosatrienoic acid (20:3n-3) to serve as substrates for Δ8 desaturase [77]. The elongase (*Elovl*) genes involved in these steps have been extensively investigated in various fish species regarding their catalytic activities and substrate specificities, as summarized in Table 6.

The coexistence of Δ6- and Δ8-dependent routes illustrates the remarkable metabolic plasticity that has evolved among aquatic organisms [33]. Species possessing both enzymatic activities may retain greater metabolic flexibility under fluctuating dietary conditions, whereas species relying on a single pathway often display narrower biosynthetic capacities [89]. The final production of DHA represents one of the most evolutionarily variable steps in LC-PUFA biosynthesis [60]. Most teleost fishes synthesize DHA through the Sprecher pathway, in which EPA undergoes sequential elongation to C24 intermediates, followed by Δ6 desaturation and peroxisomal β-oxidation to generate DHA [89]. Although effective, the Sprecher pathway involves several enzymatic steps and requires peroxisomal β-oxidation. In contrast, in some marine fish, a Δ4 desaturase can directly convert DPA to DHA, bypassing the C24 intermediates and peroxisomal chain-shortening step [90]. The discovery of functional Δ4 desaturases in multiple teleost lineages challenged the long-standing assumption that the Sprecher pathway was the universal mechanism of DHA synthesis in vertebrates [89]. These alternative pathways illustrate the diversity of mechanisms that have evolved for DHA biosynthesis [13].

Gene duplication has contributed to the diversification of LC-PUFA biosynthesis by generating additional copies of metabolic genes. In vertebrates, *Fads1* and *Fads2* originated through a tandem duplication event in the ancestral lineage [61]. Following duplication, paralogous genes can diverge functionally and develop distinct roles within metabolic pathways [91]. Conversely, species inhabiting LC-PUFA-rich marine environments frequently lost redundant biosynthetic genes because dietary availability reduced selective pressure for maintaining endogenous synthesis [92]. Such gene gain and loss illustrate a classic evolutionary trade-off between metabolic independence and energetic efficiency [93]. Maintaining complex FA biosynthesis is beneficial when dietary nutrients are limited, but it may be unnecessary when essential FAs are readily available in the diet [92]. These evolutionary processes have produced substantial differences in LC-PUFA biosynthetic capacity among aquatic animals [13,92]. Freshwater fishes generally retain greater capacity for endogenous LC-PUFA biosynthesis than many marine teleosts, a pattern associated with differences in dietary LC-PUFA availability between freshwater and marine environments [94]. Endogenously synthesized LC-PUFAs can contribute to the supply of physiologically important FAs required for growth, development, and reproduction [95].

Many marine fishes exhibit reduced biosynthetic capacity because marine food webs usually provide abundant dietary LC-PUFAs through microalgae and zooplankton [96]. Nevertheless, considerable variation exists even among marine teleosts, indicating that habitat alone does not fully determine biosynthetic potential [13]. Aquatic invertebrates display even greater diversity. Molluscs, crustaceans, echinoderms, cephalopods, and cnidarians differ markedly in their complement of desaturase and elongase enzymes [59]. Some aquatic species retain only partial LC-PUFA biosynthetic pathways, whereas others possess alternative enzymatic activities, including ωx desaturases in several invertebrate lineages and Δ4 desaturases in certain teleosts, that expand or bypass conventional biosynthetic routes [33]. Therefore, endogenous LC-PUFA biosynthesis has evolved as a continuum of metabolic strategies rather than discrete taxonomic categories [59].

Environmental conditions have further shaped the evolution of these pathways. Habitat-specific differences in primary producer communities determine the availability of dietary LC-PUFAs throughout aquatic food webs [60]. Freshwater ecosystems often contain lower concentrations of DHA than marine systems, increasing selective pressure for efficient endogenous biosynthesis [94]. Temperature can affect LC-PUFA metabolism because increasing the proportion of unsaturated FAs helps maintain membrane flexibility at low temperatures [97]. Organisms living in polar and temperate environments often have higher levels of EPA and DHA than those from warmer tropical regions, reflecting differences in lipid composition associated with temperature adaptation [98]. Similar temperature-dependent changes in PUFA composition have also been reported in freshwater organisms, including zooplankton from inland-water systems, supporting the role of homeoviscous adaptation across aquatic environments [99]. In addition, environmental factors such as salinity can influence FA composition and LC-PUFA biosynthesis in aquatic organisms [100]. Seasonal productivity and food-web structure can also modify the availability and transfer of FAs through aquatic food webs [60]. Metabolic plasticity reflects the ability of organisms to adjust their metabolic performance in response to environmental conditions, while differences in this flexibility can also have evolutionary consequences [101].

Geographical variation in FA composition can also occur among populations or strains of the same species. Such intraspecific variability may reflect differences in local environmental conditions, including temperature, salinity, nutrient availability, light, and food resources, rather than geographical location alone [60]. For example, among nine *Tetraselmis* strains isolated from the same marine locality and regarded as the same species, DHA was detected in only two strains (0.3% and 0.4% of total FAs), while ARA and GLA varied from 0.8% to 2.7% and from 0.5% to 7.3%, respectively [102]. Geographic variation has also been observed in aquatic consumers, where regional differences in environmental conditions and food-web composition can influence EPA, DHA, and other FA profiles [103]. Thus, LC-PUFA composition within a species should not be considered fixed but may vary spatially in response to local ecological and environmental conditions.

Recognizing species-specific differences in LC-PUFA biosynthetic capacity is important for developing appropriate aquaculture diets and improving the sustainability of feed formulations [100]. Species with limited endogenous LC-PUFA biosynthesis depend heavily on dietary fish oil or alternative sources of DHA and EPA [104], whereas species with stronger biosynthetic capacity can utilize plant-derived C18 fatty acids more efficiently. Knowledge of species-specific desaturase and elongase repertoires can therefore guide broodstock nutrition, feed formulation, and selective breeding programs [12]. Advances in comparative genomics, transcriptomics, gene editing, and functional enzymology are also creating opportunities to identify novel biosynthetic enzymes and improve LC-PUFA production in cultured species [105]. These approaches may reduce reliance on finite marine resources while supporting the expansion of sustainable aquaculture [16].

In summary, endogenous LC-PUFA biosynthesis has evolved through repeated cycles of innovation, diversification, and adaptation across aquatic animals [13]. The emergence of ωx desaturases, alternative front-end desaturation pathways, multiple mechanisms of DHA synthesis, and lineage-specific patterns of gene duplication and loss demonstrates that there is no single evolutionary solution for LC-PUFA production [106]. Instead, aquatic animals have evolved a wide range of metabolic strategies that reflect the interaction between genome evolution, ecological constraints, and nutritional availability. Recognizing this evolutionary and metabolic diversity provides a broader framework for understanding FA biology and offers new directions for comparative physiology, evolutionary genomics, and sustainable aquaculture research [60].

### 3.3. FA Abundance and Distribution Within Various Ecosystems

FA composition in primary producers varies substantially across ecosystems, with clear heterogeneity both within and among ecosystems. Within ecosystems, such variation is primarily associated with species differences [107] and environmental conditions [102]. The occurrence and relative abundance of EPA and DHA vary substantially among phytoplankton groups, with different taxa exhibiting distinct patterns of these LC-PUFAs. Diatoms can contain substantial amounts of EPA but generally little DHA, whereas green algae (Chlorophyceae), which are common in freshwater ecosystems, are primarily characterized by C18 PUFAs such as ALA and generally contain little or no EPA and DHA [108]. Other phytoplankton groups show distinct FA profiles, emphasizing the strong taxonomic dependence of LC-PUFA production [109,110]. Considerable variation is also observed among closely related species and among individuals within species [107], and experimental studies have demonstrated that environmental conditions, particularly temperature, nutrient availability, and light intensity, can substantially modify algal FA composition [102,111]. The distribution of EPA and DHA across aquatic ecosystems depends strongly on the taxonomic composition of primary producers. Marine food webs generally provide abundant EPA and DHA, whereas freshwater systems can also provide substantial EPA but are often comparatively limited in DHA. Since different phytoplankton taxa differ in their FA profiles, differences in phytoplankton community composition can substantially influence the content and composition of LC-PUFAs available to aquatic consumers [108,112]. FA profiles can also provide useful information on trophic pathways because particular FAs or combinations of FAs are associated with specific primary-producer groups; however, their interpretation requires caution because FAs can be selectively retained or metabolically modified by consumers [112].

Taxonomic affiliation significantly influences FA composition in both marine and freshwater organisms, alongside trophic and dietary factors [113,114]. In phytoplankton, FA profiles vary substantially among taxonomic groups and species. In consumers, FA composition reflects both dietary sources and taxon-specific metabolic characteristics. For example, studies of freshwater zoobenthic invertebrates have shown that both feeding strategy and phylogenetic affiliation contribute to variation in FA profiles [114].

In freshwater fishes, FA composition can reflect both dietary FA availability and the capacity for selective retention or endogenous biosynthesis. For example, different ecotypes of Eurasian perch (*Perca fluviatilis*) may specialize in distinct prey sources, including littoral macroinvertebrates that are relatively low in DHA and pelagic zooplankton such as copepods that can provide greater amounts of DHA. The maintenance of relatively high tissue DHA despite DHA-poor diets suggests that selective retention and endogenous DHA biosynthesis may contribute to tissue FA composition [60,115,116]. Similar dietary effects have been demonstrated in Baikal grayling (*Thymallus baicalensis*) from different river populations. Grayling consuming greater proportions of terrestrial invertebrates had lower EPA and DHA contents in muscle and adipose tissues than populations relying more strongly on aquatic food resources [48]. In addition, the detection of 24:5n-3 and 24:6n-3, intermediates of the Sprecher pathway, in Baikal grayling tissues provides indirect evidence for endogenous DHA biosynthesis in this species [117].

Terrestrial and aquatic food webs differ substantially in the FA composition and nutritional quality of their basal resources. Terrestrial plants are generally important sources of the C18 PUFAs linoleic acid and α-linolenic acid, whereas aquatic primary producers can provide greater amounts of LC-PUFAs such as EPA and DHA. These differences are subsequently reflected in the FA composition of consumers. For example, terrestrial phytophagous invertebrates can contain relatively high levels of LIN and/or ALA but generally low levels of EPA and ARA, whereas aquatic insects can provide substantially greater amounts of EPA. However, terrestrial predators and scavengers may accumulate EPA and ARA through consumption of aquatic-derived prey, demonstrating that PUFA transfer across the aquatic–terrestrial boundary can occur through multiple trophic pathways [118]. The nutritional differences between aquatic and terrestrial prey can also influence terrestrial vertebrate consumers. For example, access to emergent aquatic insect prey varies markedly among tree swallow populations, with aquatic insects providing substantially higher concentrations of EPA than terrestrial food sources. Experimental diet studies indicate that Tree Swallow nestlings are relatively inefficient at synthesizing EPA and DHA from the precursor ALA [119]. Greater consumption of aquatic insects or diets enriched in EPA and DHA promotes faster growth, improves body condition, and enhances survival in Tree Swallow chicks [120]. Nest sites differ greatly in their distance from aquatic habitats. As a result, adult Tree Swallows may face a trade-off between prey quality and prey quantity when feeding their chicks. This reliance on cross-ecosystem subsidies highlights the traditional view that terrestrial consumers depend on aquatic food webs for preformed LC-PUFAs. Although aquatic-derived resources represent an important source of LC-PUFAs for terrestrial food webs, some soil-litter invertebrates may also provide nutritionally important LC-PUFAs to terrestrial consumers. For example, Kuznetsova et al. [121] reported high EPA contents in hygrophilous springtails (Collembola) with different diets and habitats, indicating that these abundant terrestrial arthropods may represent an important potential source of EPA for higher trophic-level terrestrial consumers.

In some cases, habitat-dependent variation in FA composition may contribute to evolutionary divergence in consumers. Evidence from Great Tits (*Parus major*) shows that urban and rural populations differ in their feeding habits [122], FA composition [122,123], and the expression of genes involved in LC-PUFA biosynthesis, including *Elovl* and *Fads* pathways [124], which code for the enzymes used to convert ALA and LIN to n-3 and n-6 LC-PUFAs, respectively. The finding that urban Great Tits actively downregulate LC-PUFA biosynthetic enzymes presents an intriguing evolutionary puzzle: if n-3 fatty acids are indispensable, why would city-dwelling birds suppress their production? One plausible explanation is that the physiological costs, particularly heightened vulnerability to oxidative damage, offset any benefits in environments where inflammation and oxidative stress already impose fitness penalties, thereby challenging the notion that enhanced biosynthesis is always advantageous [122,123,124,125].

### 3.4. Bidirectional Flux of Fatty Acids Across Aquatic-Terrestrial Ecosystems

The exchange of nutrients and biomolecules across aquatic-terrestrial boundaries is bidirectional and plays an important role in linking adjacent food webs. Aquatic ecosystems can export LC-PUFAs to terrestrial consumers through vectors such as emerging aquatic insects and animals moving between aquatic and terrestrial habitats [126,127,128,129]. Conversely, terrestrial organisms and organic matter can transfer FAs back into aquatic ecosystems, providing allochthonous nutritional subsidies to aquatic consumers. Quantitative evidence from a shaded river demonstrated that the import of terrestrial invertebrate biomass into the aquatic ecosystem exceeded the export of emerging aquatic insects and represented a substantial flux of both n-3 and n-6 PUFAs [47]. Importantly, these reciprocal fluxes differed in nutritional quality: terrestrial invertebrate inputs supplied proportionally less n-3 PUFA but more n-6 PUFA than aquatic invertebrate resources [47]. More recent work has further identified the terrestrial invertebrate groups involved in this return flux, showing that phytophagous insects can supply LIN and ALA, whereas terrestrial predators and scavengers can return EPA and ARA of aquatic origin to the river through a “boomerang” pathway [118].

Many terrestrial animals have a limited capacity to synthesize LC-PUFAs from shorter-chain precursors such as ALA, particularly because of constraints in the conversion of ALA to EPA and DHA, resulting in a strong reliance on dietary sources of these LC-PUFAs. This difference in LC-PUFA availability and biosynthetic capacity originates at the base of food webs and can influence consumers across trophic levels. Aquatic and terrestrial organisms generally differ in their FA composition, particularly in their LC-PUFA content [49]. Despite their recognized importance for neural and ocular tissues, development, and seasonal physiological demands, the overall quantitative requirement for LC-PUFAs across all terrestrial animals remains incompletely understood [49,130,131].

Freshwater systems, especially wetlands, serve as critical sources of LC-PUFAs for land-based food webs through both direct consumption of aquatic prey and indirect pathways [2,49]. This transfer is facilitated by animals with dual aquatic-terrestrial life stages, such as insects and amphibians, as well as predators that forage on aquatic resources [132]. Emerging aquatic insects represent one of the primary conduits for moving LC-PUFAs and organic matter from water to land [128,133]. Species belonging to Ephemeroptera, Plecoptera, Trichoptera, and some Diptera develop in aquatic environments and emerge as adults that become prey for riparian spiders, birds, and bats [134].

These insects typically contain substantially higher amounts of C20 and C22 PUFAs than terrestrial insects, often by an order of magnitude [132]. Emergence is often seasonal, influencing the growth, biomass, and development of terrestrial predators [135], with stable isotope analyses confirming significant nutritional contributions [136]. Agricultural activities, including nutrient loading, pesticide application, and habitat alteration, modify aquatic insect communities by changing species composition and biomass [137]. These changes can reduce the availability of LC-PUFA-rich prey for riparian predators, affecting spider diets, richness, and abundance [138,139]. The impacts of agricultural intensification on LC-PUFA subsidies depend on pollutant type, insect taxa affected, and timing, highlighting the need for context-specific conservation approaches. Experiments indicate that intermediate nutrient levels may maximize FA export by midges, while some toxicants show limited direct effects on adult insect FA composition [140,141].

Further research is required to clarify mechanisms behind reduced PUFA levels in insects from agricultural landscapes. Effective conservation should prioritize reduced pesticide use and riparian reforestation to improve habitat quality and sustain these nutritional subsidies [142]. For many omnivorous and carnivorous terrestrial consumers, dietary quality, particularly LC-PUFA content, often matters more than total food quantity [120]. Fluctuations in the timing and magnitude of these subsidies can exert selective pressure on species in LC-PUFA-limited environments [143]. Amphibians function as significant conduits for transferring LC-PUFAs from aquatic to terrestrial ecosystems. The magnitude of these subsidies varies among species and wetlands [144]. Direct measurements of amphibian-mediated LC-PUFA fluxes have shown that the export of EPA and DHA differs among amphibian species and ponds, with anurans generally exporting greater amounts of LC-PUFAs than salamanders, largely because of their greater emergence biomass [144]. Larger ponds may export greater absolute amounts of EPA and DHA, whereas some smaller ponds can produce relatively high exports per unit area. In parallel, studies based on amphibian abundance and biomass demonstrate that biomass export is strongly influenced by species identity and community composition [145]. Thus, both the taxonomic composition of emerging amphibian communities and the magnitude of their biomass export can influence the transfer of high-quality resources from aquatic to terrestrial ecosystems. Because numerous terrestrial predators feed on amphibians, these cross-habitat subsidies represent a potentially important but still incompletely understood resource [146]. Even small ponds can generate meaningful fluxes relative to their surface area. Human-driven changes are likely to alter amphibian-mediated transfers of LC-PUFAs and thereby influence adjacent terrestrial food webs [147]. Further work is needed to evaluate how wetland attributes such as vegetation structure, primary productivity, and canopy cover, together with the contribution of juvenile amphibians to terrestrial food webs, govern the magnitude and ecological significance of these transfers [148].

### 3.5. Availability of n-3 LC-PUFA and Habitat Selection

Differences in n-3 LC-PUFA availability among habitats can create contrasting nutritional conditions, potentially influencing how aquatic consumers obtain these FAs through their diets or through endogenous biosynthesis. Limited access to DHA may consequently contribute to variation in LC-PUFA biosynthetic capacity, which could affect ecological performance and habitat use. Ecologically underused or vacant niches may provide opportunities for divergent selection and subsequent diversification of lineages [149]. Because the supply of n-3 LC-PUFAs differs considerably among food webs, variation in access to these nutrients can shape consumer behavior, dietary choices, and ecological interactions [120]. The colonization of habitats containing previously underexploited resources can likewise create conditions conducive to adaptive radiation [150]. Nevertheless, ecological opportunity does not invariably result in diversification across all evolutionary lineages. Repeated shifts from marine environments into freshwater have been associated with diversification in fishes, although pronounced radiations have occurred primarily in certain lineages [151]. The causes of this variation at physiological and genetic levels remain unresolved.

A frequently overlooked constraint on marine-to-freshwater colonization is the low nutritional value of freshwater food resources [152], which is crucial for animal health and development [10]. The set of limiting nutrients includes LC-PUFAs, especially DHA. Primary producers in marine ecosystems generally have greater EPA and DHA content than those in freshwater ecosystems, which are richer in the precursor ALA. For example, in marine ecosystems with an incomplete capacity for DHA biosynthesis, marine Acanthopterygii obtain sufficient DHA from their natural diet. In freshwater systems, however, DHA is scarce in prey resources. The base-level producers in the freshwater food webs are marked by elevated ALA content but low DHA availability [60]. This nutritional limitation may be further reinforced in shaded streams where fish rely substantially on terrestrial invertebrate inputs. Recent evidence indicates that terrestrial phytophagous invertebrates entering such streams primarily provide the C18 PUFAs LIN and ALA, while containing relatively low amounts of preformed LC-PUFAs [118].

Freshwater teleosts belonging to the ancient Osteoglossomorpha lineage and the highly diverse Otophysi lineage retain the capacity for endogenous DHA biosynthesis, reflecting an evolutionary adaptation to freshwater environments where dietary DHA is often limited. This is consistent with the notion that limited availability of DHA in freshwater food webs is thought to have favored the evolution and maintenance of endogenous DHA biosynthesis, indicating that dietary DHA alone may be insufficient for normal development and survival. Despite this nutritional constraint, some marine Acanthopterygii, which historically relied entirely on exogenous DHA, have successfully adapted to and colonized freshwater ecosystems [94].

The traditional emphasis on osmoregulation as the primary barrier to freshwater colonization may overlook other important adaptations. The independent duplication of *Fads2* genes across multiple freshwater lineages suggests that enhanced DHA biosynthesis may be equally crucial for survival in new environments, challenging existing views of the physiological demands associated with aquatic habitat transitions [152]. In addition to osmoregulatory adaptation, Acanthopterygii colonizing freshwater ecosystems faced another major obstacle: the imbalance between their physiological requirements for DHA and the restricted dietary supply available in freshwater food webs [94]. Spatial differences in the environment create different selective pressures, which can lead to two possible outcomes: organisms may rely more on dietary DHA intake, or they may evolve a stronger capacity to produce DHA from precursor FAs within their own bodies [60].

In fish, multiple lineages that have colonized DHA-deficient freshwater habitats exhibit a convergent duplication of the *Fads2* gene, a key gene involved in DHA biosynthesis, indicating that increased *Fads2* copy number may represent an adaptation to DHA-deficient freshwater habitats [153]. Stream populations generally possessed higher *Fads2* copy numbers than lake populations, suggesting that habitat-specific dietary differences can drive divergent selection on LC-PUFA biosynthetic capacity. Benthic-associated populations also exhibited elevated *Fads2* copy numbers compared with planktivorous populations, indicating that adaptation to benthic feeding may increase the selective pressure for enhanced DHA biosynthesis. Moreover, fish consuming DHA-poor terrestrial prey, such as insects, showed higher *Fads2* copy numbers, whereas populations feeding on DHA-rich prey, including copepods and other aquatic organisms, displayed lower copy numbers [152]. These findings suggest that spatial variation in dietary LC-PUFA availability may act as an important selective force shaping adaptive diversification and the evolution of LC-PUFA biosynthetic routes in aquatic animals [154].

Spatial differences in prey availability between lake and stream environments may promote divergent selection, leading to variation in *Fads2* copy number. The association between *Fads2* copy number and DHA availability challenges a simple interpretation of adaptation. Increased gene dosage may provide an advantage in DHA-limited environments, yet become unnecessary or costly under DHA-rich conditions. Thus, this trait may represent ecological specialization rather than a general evolutionary improvement [154]. In addition to increasing gene dosage, *Fads2* duplications may facilitate the evolution of new functional roles [155]. This genetically mediated variation in DHA acquisition capacity has consequences that extend beyond physiology because *Fads2* copy number tracks habitat-specific dietary DHA availability; it can also constrain or enable which habitats a population is able to successfully exploit during diversification. This link is illustrated by adaptive radiations in salmonids, where habitat selection along a pelagic-littoral axis closely tracks n-3 LC-PUFA availability. The ecological consequences of variation in LC-PUFA acquisition and biosynthetic capacity may extend beyond physiological adaptation and influence habitat use during diversification events [154,156]. Postglacial European whitefish (*Coregonus* spp.) provide a well-documented example of rapid ecological diversification, with populations evolving distinct littoral, pelagic, and profundal forms associated with differences in habitat use and trophic resources [157]. Similar patterns of ecological divergence have evolved repeatedly in Arctic charr (*Salvelinus alpinus*), where sympatric ecotypes show differences in feeding ecology and associated morphological and gene-expression traits across independent populations [158]. These examples demonstrate how contrasting ecological opportunities within freshwater environments can promote repeated phenotypic divergence and the evolution of distinct ecological niches.

Pelagic and littoral habitats can impose contrasting nutritional and ecological conditions that contribute to the diversification of freshwater fishes. In Arctic charr, for example, ecological divergence along depth gradients has produced distinct morphs associated with pelagic, littoral, and profundal habitats, including planktivorous, dwarf, piscivorous, and abyssal forms [159]. Such diversification can be influenced by interactions among prey availability, intraspecific competition, and predation, which alter the selective environment experienced by different phenotypes. More broadly, habitat-specific differences in resource availability can promote ecological specialization, although the contribution of LC-PUFA biosynthetic capacity to these diversification processes remains insufficiently resolved. [160,161]. However, whether variation in LC-PUFA biosynthetic capacity contributes directly to such diversification remains insufficiently understood. The ecological consequences of diversification can extend beyond the focal lineage, as changes in predator phenotypes and habitat use may propagate through food webs and modify interactions with prey and other community members [162]. Conversely, diversification within a food-web component can alter predator–prey interactions and potentially generate cascading evolutionary and ecological effects across trophic levels. Changes in prey diversity and ecological characteristics can consequently modify the selective environment experienced by predators, potentially promoting divergence in their foraging strategies and phenotypes [163].

### 3.6. Mechanisms Regulating LC-PUFA Biosynthesis

LC-PUFA biosynthesis in fish proceeds through a series of desaturation and elongation reactions that convert C18 PUFAs, namely, linoleic acid (LIN, 18:2 n−6) and α-linolenic acid (ALA, 18:3 n−3), into long-chain products, such as ARA, EPA, and DHA [164]. The following main steps have been investigated in studies of the transcriptional regulation of LC-PUFA biosynthesis: (1) cloning and characterization of *Fads2* and *Elovl* promoters, (2) identification of the key transcription factor modulating *Fads2* and *Elovl* target gene expression, and (3) verification of transcriptional regulatory functions and mechanisms of transcription factors in LC-PUFA biosynthetic pathways [12]. In the LC-PUFA biosynthetic pathway, the corresponding genes encode elongases and desaturases whose expression and activity influence LC-PUFA biosynthesis [12]. In fish, Δ6 fatty acyl desaturase (Fads2) has been extensively studied as it is regarded as the rate-limiting enzyme in the LC-PUFA biosynthetic pathway [165]. Moreover, LC-PUFA biosynthetic activity is subject to regulation by environmental cues that can affect pathway dynamics [166].

#### 3.6.1. Transcriptional Regulation

Transcription factors (TFs), referred to as trans-acting regulatory proteins, modulate gene expression by interacting with cis-acting elements located in gene promoter regions. Depending on their function as activators or repressors, they influence the recruitment and activity of RNA polymerase during transcription initiation. The promoter region is classically defined as the upstream non-coding sequence of a gene, extending from the transcription start site (TSS), which is assigned position +1 in most promoter studies [12].

LC-PUFA biosynthetic genes such as *Fads2* and *Elovl* are under the control of major transcription factors linked to lipid metabolic regulation. In particular, the Lxrα–Srebp-1c pathway contributes to LC-PUFA biosynthesis in the hepatopancreas by mediating transcriptional activation of *Elovl5* as well as *Fads2* [167]. A variety of transcription factors participate in the regulation of LC-PUFA biosynthesis, including peroxisome proliferator-activated receptors (PPARs), sterol regulatory element-binding proteins (SREBPs), liver X receptor (LXR), and hepatocyte nuclear factor 4α (HNF4α) [168]. Regulation of LC-PUFA biosynthesis in teleosts is mediated by a complex network of lipid metabolism-related transcription factors that control key desaturase genes. For instance, transcriptional activation of *Fads2* is influenced by Srebp-1 and Ppar-α in Japanese sea bass (*Lateolabrax japonicus*) [169]. In contrast, in herbivorous marine species such as rabbitfish (*Siganus canaliculatus*), Hnf4α has been proposed to act as an upstream regulator enhancing expression of the bifunctional Δ5/Δ6 *Fads2* gene. An outstanding unresolved issue is whether interspecific variation in transcriptional regulation represents ecologically driven adaptation or effectively neutral evolutionary drift; the scarcity of in vivo experiments conducted under environmentally realistic conditions prevents us from discriminating between these alternatives [170].

The capacity for LC-PUFA biosynthesis in fish is determined not only by the repertoire of desaturase and elongase genes and their enzymatic activities, but also by the regulation of gene expression at both transcriptional and post-transcriptional levels [171]. Key transcriptional regulatory pathways of LC-PUFA biosynthesis, including Lxrα–Srebp-1 signaling, are conserved between teleosts and mammals. However, teleost fishes exhibit a more complex regulatory landscape, likely due to an expanded repertoire and greater functional diversification of *Fads2* and, to a lesser extent, *Elovl* genes [61].

Studies in zebrafish have shown that transcriptional control of LC-PUFA biosynthesis involves multiple promoter elements in *Fads2*, including sterol regulatory elements (SRE), Sp1, NF-Y, and CCAAT motifs [172]. *Srebp-1* overexpression in zebrafish liver cells (ZFL; ATCC^®^ CRL-2643™) enhanced Δ6 and Δ5 *Fads2* and *Elovl5* promoter activities, further confirming its role in regulating LC-PUFA biosynthesis in *Danio rerio* [12]. Feeding experiments in juvenile Golden pompano indicate a very limited or absent ability to biosynthesize LC-PUFAs from C18 PUFA precursors [173]. Furthermore, full-length mRNAs encoding two candidate Elovl elongases and two candidate Fads2 desaturases were isolated, and their functional activities were assessed via heterologous yeast expression systems [174]. The two putative elongases possess Elovl4 and Elovl5 activities. Meanwhile, both Fads2 have been reported to display Δ8/Δ5/Δ4 desaturase activities [175]. Promoter analyses of *Elovl5*, *Elovl4*, and *Fads2* have shown that Pparαb functions as a key positive transcriptional regulator of these genes, consistent with regulatory patterns observed across other teleosts and vertebrates [12]. Dietary LC-PUFA levels have been shown to exert a regulatory influence on lipid metabolism-related gene expression in large yellow croaker. Both in vivo feeding trials and in vitro hepatocyte culture studies demonstrated that elevated dietary LC-PUFA suppress the expression of key genes, including *Lxrα*, *Srebp-1*, Δ6 *Fads2*, *Elovl5*, and *Elovl4* [83]. These findings indicate a potential regulatory interaction between Lxrα, Srebp-1, and core LC-PUFA biosynthetic genes in this species. Accordingly, the full-length cDNAs and promoter regions of Δ6 *Fads2*, *Elovl5*, and *Elovl4* were subsequently cloned and characterized to further investigate their transcriptional regulation [176].

Functional assays in HEK 293T cells demonstrated that overexpression of *Lxrα* significantly enhanced the promoter activities of *Elovl4* and *Elovl5*. In contrast, *Srebp-1* overexpression reduced the promoter activities of Δ6 *Fads2* and *Elovl4*, indicating distinct regulatory effects of these transcription factors on LC-PUFA biosynthetic genes. Consistent with these findings, experiments in primary hepatocytes showed that pharmacological activation of Lxrα upregulated *Elovl4* expression, whereas inhibition of Srebp-1 resulted in its downregulation. *Elovl5* expression was also induced by Lxrα activation but remained unaffected by Srebp-1 inhibition. Collectively, these results confirm that *Elovl4* transcription is jointly regulated by Lxrα and Srebp-1 within the LC-PUFA biosynthetic pathway [169].

#### 3.6.2. MiRNA Regulation of LC-PUFA Biosynthetic Genes

In eukaryotes, gene expression is not limited to transcriptional control but is also subject to post-transcriptional regulation. Following transcription, mRNAs are extensively processed and regulated through export, localization, degradation, and translation, which collectively constitute essential components of gene expression [12]. Recent developments in high-throughput sequencing technologies and computational prediction tools have led to the discovery of multiple classes of noncoding RNAs (ncRNAs). In association with protein effector complexes, these ncRNAs regulate target mRNA expression through post-transcriptional mechanisms, including translational repression [177]. MicroRNAs (miRNAs) are key post-transcriptional regulators that control gene expression by either inducing degradation of target mRNAs or repressing their translation [178].

miRNAs are small noncoding RNA molecules, typically ~22 nucleotides in length, that mediate post-transcriptional regulation of gene expression [179,180]. They exert their regulatory effects by interacting with specific mRNA targets, resulting in either inhibition of translation or promotion of mRNA degradation [181]. The regulatory involvement of miRNAs in LC-PUFA biosynthesis has been widely explored in *Siganus canaliculatus*. The predominant reliance on rabbitfish for miRNA regulation studies represents a significant limitation, compromising the extrapolation of findings across phylogenetically diverse taxa; consequently, it remains uncertain whether these regulatory mechanisms represent conserved vertebrate traits or rabbitfish-specific adaptations [182]. The discovery of miRNAs began in zebrafish (*Danio rerio*) in 2003, where early computational tools such as miRscan were developed to predict miRNA candidates across different developmental stages and tissues [183]. Since then, the identification of miRNAs has expanded to numerous economically important fish species, broadening their known distribution in aquatic organisms [184]. Early studies on fish microRNAs (miRNAs) focused on their cloning and identification across a range of species during development. Ramachandra et al. (2008) reported miRNAs associated with early embryonic development in rainbow trout (*Oncorhynchus mykiss*) [185]. Subsequent studies extended miRNA identification to other economically important species, including common carp (*Cyprinus carpio*) [186], channel catfish (*Ictalurus punctatus*) in 2013 [187], flounder (*Paralichthys olivaceus*) in 2011 [188], and the large yellow croaker (*Larimichthys crocea*) in 2014 [184], along with initial insights into their potential regulatory roles. Overall, these studies primarily aimed to discover novel miRNAs across different fish species, tissues and organs, developmental stages, and experimental conditions.

After 2015, research increasingly focused on the detailed characterization of miRNA expression profiles [189,190]. Additionally, miRNA microarrays (miRNA chips) were employed to monitor changes in miRNA expression and identify differentially expressed miRNA profiles [189]. Current evidence indicates that miRNA research in fish has largely centered on their regulatory roles in long-chain FA biosynthesis, cholesterol metabolism, and the control of glucose and lipid metabolic pathways [191]. The rapid development of miRNA-based applications has outpaced our understanding of their complex regulatory networks. Since individual miRNAs can influence numerous target transcripts, their manipulation may generate unpredictable downstream effects. Addressing this knowledge gap through systems-level analysis is crucial for ensuring the safe and precise use of miRNA technologies [191]. The transcriptional regulation of LC-PUFA biosynthesis has been extensively investigated in Atlantic salmon, a teleost species with one of the most comprehensively characterized complements of enzymes responsible for this biosynthetic pathway [12]. Among these, miR-17 and miR-146a have been reported to directly target key desaturase and elongase genes involved in LC-PUFA biosynthesis, whereas miR-24, miR-33, miR-26a, miR-145, and the miR-15/16 cluster regulate this pathway indirectly by targeting transcription factors that control the expression of these genes [192]. In rabbitfish (*Siganus canaliculatus*), miR-17 has been reported to modulate LC-PUFA biosynthesis through repression of hepatic Δ4 Fad expression [193]. LC-PUFA biosynthesis is negatively regulated by miR-17 and miR-146a, which suppress the hepatic expression of Δ4 *Fad2* and *Elovl5*, respectively [194].

#### 3.6.3. Ecological and Environmental Drivers

In aquatic ecosystems, lipids and their fatty acid components represent not only major energy sources for consumers but also reliable biomarkers for tracing organic matter dynamics within food webs [195]. Aquatic food webs are largely supported by primary production from pelagic phytoplankton, benthic periphyton, and macrophytes, which provide energy and nutrients to secondary consumers. However, the balance between pelagic and benthic production differs markedly across ecosystems and is regulated by environmental variables [196,197].

Eutrophication is characterized by elevated nutrient levels, primarily nitrogen and phosphorus, in aquatic ecosystems. These nutrient inputs stimulate excessive algal growth, leading to declines in water quality and disruptions to ecosystem functioning [198]. Eutrophication can stimulate pelagic phytoplankton proliferation, which in turn suppresses benthic primary production by limiting light availability, depleting nutrients, and inducing hypoxia in deeper waters. As a consequence, the relative contribution of benthic production declines, and aquatic food webs become more reliant on pelagic primary production [196]. Alterations in phytoplankton community structure can significantly modify the availability of ω-3 fatty acids, reflecting the unequal distribution of PUFA biosynthetic capacity across phytoplankton taxa [107]. As reported across nutrient gradients [199], eutrophication has been shown to alter phytoplankton composition, leading to a reduction in EPA- and DHA-rich taxa. Changes in the FA composition of primary producers can propagate through aquatic food webs; however, consumer FA profiles do not simply reflect those of their dietary sources. Aquatic consumers can selectively retain physiologically important FAs and modify dietary FAs through metabolic processes. Consequently, consumer FA composition reflects the combined effects of dietary availability, selective retention, and metabolic modification [195].

Heavy-metal pollution represents another environmental stressor that can influence LC-PUFA dynamics in aquatic ecosystems. Trace metals play important roles in phytoplankton metabolism, and their availability can influence phytoplankton growth and community structure [200]. Exposure to heavy metals can also affect FA metabolism in aquatic animals. In fathead minnow (*Pimephales promelas*), exposure to cadmium (Cd) and nickel (Ni) altered membrane FA composition and affected the transcription of desaturase and elongase genes involved in FA metabolism [201]. More directly, in silver pomfret (*Pampus argenteus*), Cd exposure affected the regulation of LC-PUFA biosynthesis-related genes and reduced DHA concentration in the brain [202]. These findings indicate that metal contamination can influence FA metabolism in aquatic organisms and may consequently modify LC-PUFA availability and nutritional quality within aquatic food webs.

Phytoplankton community structure and FA composition are sensitive to environmental conditions. In particular, increasing water temperature can reduce the proportion and production of n-3 LC-PUFAs in phytoplankton, potentially decreasing the availability of EPA and DHA at the base of aquatic food webs [54]. Apart from high-upwelling regions, climate warming can alter ocean nutrient distributions, reducing nutrient availability in surface waters and marine primary productivity [203]. These changes, together with the direct effects of warming on phytoplankton FA composition, may reduce the production and availability of n-3 LC-PUFAs in marine food webs [54]. Alterations in phytoplankton community composition and lipid nutritional value can subsequently affect energy and nutrient transfer to higher trophic levels. These changes may also impair growth and reproductive efficiency in higher trophic organisms [204]. Collectively, these changes intensify biomass loss at higher trophic levels, commonly described as trophic amplification. Furthermore, the rising occurrence of mild winters in the Mediterranean has promoted small phytoplankton groups, such as cyanobacteria (<1 μm), picoeukaryotes (<1 μm), and nanoeukaryotes (3–6 μm), while concurrently decreasing larger phytoplankton, particularly diatoms, during spring blooms [205]. Key climate change drivers, including ocean warming and acidification, can modify the lipid nutritional quality of marine organisms [206]. In *Dicathais orbita*, exposure to future ocean scenarios (25 °C, 750 ppm pCO_2_) resulted in a substantial decrease in foot tissue lipid content (~3 mg/g) compared with organisms kept under present-day conditions (23 °C, 379 ppm pCO_2_; ~6 mg/g). However, the overall FA composition remained largely unchanged, being dominated by PUFAs and maintaining an n-3:n-6 ratio close to 2 [206]. Consequently, climate-driven reductions in the production and availability of n-3 LC-PUFAs in aquatic ecosystems may reduce their supply to human diets, potentially exacerbating inadequate dietary intake of these essential FAs [207].

Stable isotope techniques, including ^13^C-labeling and fatty acid-specific isotopes, may be used to trace the production of high-quality PUFAs that are subsequently consumed by selective grazers [208]. Evaluating the dietary quality of aquatic organisms based on their PUFA composition is an increasingly important approach for understanding somatic growth, reproductive success, and survival of aquatic consumers [209]. Aquatic animals are incapable of de novo synthesis of essential fatty acids (EFAs) [120], and possess limited capacity to convert these precursors into LC-PUFAs, including ARA, EPA, and DHA, which are fundamental for somatic growth, development, and reproduction in aquatic consumers [210].

The observation that tropical eutrophic systems sustain fish populations without evident LC-PUFA deficiency fundamentally questions the established relationship between ecosystem productivity and nutritional quality, implying either that unrecognized LC-PUFA sources exist or that tropical species possess enhanced biosynthetic capacities compared to temperate counterparts [211]. These findings suggest that climatic differences can significantly influence FA retention patterns in consumers within eutrophic ecosystems [211], and may contrast with the lower LC-PUFA retention observed in consumers from mainly temperate aquatic systems [212]. The prevailing assumption that EPA and DHA concentrations decrease as eutrophication intensifies in aquatic ecosystems warrants further refinement. Although the relative concentrations of these LC-PUFAs (per unit of sestonic organic carbon or fish biomass) tend to decline, the total production, including the amount available for human consumption, expressed as LC-PUFA per unit area or volume per unit time, does not necessarily decrease. While mesotrophic waters are regarded as optimal for LC-PUFA provisioning to humans, this perspective overlooks confounding variables including production efficiencies and harvesting practices, raising fundamental questions about whether ecosystem-based governance can successfully reconcile biodiversity conservation priorities with human nutritional requirements [4].

## 4. Future Trends and Perspectives

Our understanding of LC-PUFA biosynthesis has progressed from the identification of the core Fads and Elovl enzymes toward a more complex view in which gene repertoire, enzyme function, regulatory mechanisms, and physiological context interact to determine biosynthetic capacity [12]. Comparative studies have shown that the number and type of *Fads* and *Elovl* genes vary among vertebrates, while functional studies in teleosts have revealed substantial differences in substrate specificity and enzymatic activity [13,213,214]. Future research should therefore move beyond the identification of individual biosynthetic genes and focus on how *Fads* and *Elovl* repertoires evolve, how duplicated enzymes acquire distinct functions, and how their activities are coordinated across tissues, developmental stages, and environmental conditions.

Gene duplication and loss are important mechanisms underlying the evolutionary diversification of LC-PUFA biosynthesis [13]. Studies in freshwater fish have shown that changes in *Fads2* copy number can contribute to differences in LC-PUFA biosynthetic capacity among lineages and populations [152,154,215]. Recent population-level evidence further indicates that multiple freshwater stickleback populations across Europe and elsewhere have accumulated *Fads2* copy-number duplications associated with adaptation to diverse nutritional landscapes [153]. In addition, molecular and functional characterization of *Elovl2* in Chinese sturgeon demonstrated the evolutionary conservation of this elongase and its important role in LC-PUFA biosynthesis [78]. Future comparative studies should therefore determine how changes in *Fads* and *Elovl* gene dosage arise across lineages and whether duplicated genes undergo functional specialization, subfunctionalization, or retention of partially redundant activities [152].

Functional characterization also demonstrates that gene annotation alone cannot fully predict LC-PUFA biosynthetic capacity. Different *Fads* and *Elovl* paralogs can exhibit distinct desaturase or elongase activities and substrate preferences [213,214]. Future studies should therefore combine comparative sequence analysis with enzyme characterization, tissue-specific expression, and substrate utilization assays to determine how individual paralogs contribute to alternative biosynthetic routes. This approach will be particularly important for species in which gene duplication, loss, or functional diversification has altered the conventional LC-PUFA biosynthetic pathway [13,78,214].

Regulation represents another major area for future research. Transcriptional and post-transcriptional mechanisms involving *Lxr*, *Srebp*, *Hnf4α*, *Ppar*, *Sp1*, and microRNAs have been implicated in the regulation of *Fads* and *Elovl* genes in teleosts [12,216]. For example, *Pparαb* has been shown to activate *Fads2* transcription in golden pompano, providing direct evidence that transcription factors can regulate LC-PUFA biosynthetic genes [174]. Environmental conditions may also modify this regulation; recent work showed that salinity modulated the expression of LC-PUFA biosynthesis-related genes, particularly *Fads2*, in a euryhaline flatfish [217]. Future studies should therefore clarify how transcriptional, post-transcriptional, and environmental signals interact across tissues and developmental stages to regulate LC-PUFA biosynthesis [12,216,217].

Genomic studies are increasingly identifying genetic variants associated with FA composition, including candidate regions near *Elovl2* and *Fads2* in Atlantic salmon [218,219]. However, genetic association does not by itself establish a direct role in LC-PUFA biosynthetic control. Functional genetic approaches can help establish causality, as demonstrated by CRISPR/Cas9-mediated disruption of *Elovl2* in zebrafish, which resulted in a marked reduction in endogenous DHA biosynthesis [220]. Future research should extend such approaches to additional *Fads* and *Elovl* paralogs and combine genetic manipulation with enzyme assays and quantitative lipidomic analyses. Such integrated approaches will help distinguish genes that are associated with LC-PUFA traits from those that directly control biosynthetic activity [12,13].

Overall, the next stage of LC-PUFA research should shift from cataloguing *Fads* and *Elovl* genes toward a mechanistic understanding of how these pathways evolve, function, and respond to biological and environmental changes [12,13]. Integrating comparative genomics, functional characterization of enzymes, regulatory analysis, and functional genetic validation should clarify how genetic and regulatory variation translates into differences in LC-PUFA biosynthetic capacity among species. Such an integrated framework will help resolve the remaining links between gene evolution, enzyme function, pathway regulation, and actual LC-PUFA production.

## 5. Conclusions

This review combines extensive bibliometric analyses with a detailed thematic review to provide an overview of LC-PUFA biosynthesis in aquatic animals. An analysis of 1083 Scopus-indexed publications (1991–2025) showed a clear increase in publication output on LC-PUFA biosynthesis over the past three decades. Early studies (1991–2005) were limited by available molecular tools. Publication output increased after 2006, coinciding with advances in genome sequencing and an expanding focus on nutritional and adaptive mechanisms. A further increase occurred after 2015, with publication output reaching its highest level in 2020, alongside growing research interest in sustainable ω-3 sources and emerging molecular and gene-editing technologies. Although annual output has stabilized at 60–75 papers, the field demonstrates sustained research activity and increasing international participation, with major contributions from China, the UK, Spain, the USA, and Australia. Collaborative networks have also provided a tightly connected global research community. The keyword co-occurrence map highlights emerging research focus areas in metabolomics, environmental regulation, microalgae-derived ω-3 sources, and gene editing, signaling a shift toward integrative and technology-driven research.

This thematic review highlights the central role of microalgae and other lower trophic-level organisms as the primary sources of LC-PUFAs in water-based ecosystems and emphasizes how the transfer of these essential FAs through food webs shapes the nutritional quality of aquatic and adjacent terrestrial environments. The distribution and availability of LC-PUFAs vary substantially among ecosystems, taxa, and habitats, driving differences in trophic interactions, aquatic–terrestrial FA fluxes, and ecological specialization. Variation in dietary LC-PUFA availability has also shaped the evolution of endogenous biosynthetic capacity, with lineage-specific changes in desaturase and elongase repertoires enabling aquatic animals to adapt to contrasting nutritional environments. LC-PUFA biosynthesis is further modulated by transcriptional and post-transcriptional mechanisms, including transcription factors and microRNAs, allowing pathway activity to respond to nutritional and environmental conditions. Collectively, current evidence indicates that LC-PUFA dynamics arise from the interplay between primary production, trophic transfer, endogenous biosynthetic capacity, and environmental drivers, underscoring their fundamental importance in ecosystem functioning, evolutionary adaptation, and sustainable aquaculture development.

Future progress will depend on moving beyond the identification of *Fads* and *Elovl* genes toward establishing the mechanisms that determine actual LC-PUFA biosynthetic capacity. Comparative genomics, functional characterization of enzymes, regulatory analyses, lipidomics, and functional genetic approaches should increasingly be integrated to determine how genetic variation affects enzyme function, how biosynthetic pathways are regulated across tissues and environmental conditions, and which genes directly control LC-PUFA production. Such integration should provide a more mechanistic understanding of how LC-PUFA biosynthetic pathways evolve, function, and adapt across aquatic animals.

## Figures and Tables

**Table 1:** Distribution of document types.

Document Type	No. of Documents	Percentage (%)
Article	1005	92.8
Review	46	4.25
Book Chapter	9	0.83
Conference Paper	5	0.46
Editorial	5	0.46
Short Survey	4	0.37
Erratum	3	0.28
Data Paper	2	0.18
Note	2	0.18
Book	1	0.09
Letter	1	0.09

**Table 2:** Top 10 highly cited documents.

Rank	Authors	Title	Journal	Country	Citations	Journal IF	Year	Document Type
1	LFC Castro, DR Tocher, O Monroig [13]	“Long-chain polyunsaturated fatty acid biosynthesis in chordates: Insights into the evolution of Fads and Elovl gene repertoire”	Progress in Lipid Research	Portugal	364	14.9	2016	Review
2	N Hastings, M Agaba, DR Tocher et al. [14]	“A vertebrate fatty acid desaturase with Δ5 and Δ6 activities”	PNAS	UK	308	9.1	2001	Article
3	RJ Henderson [32]	“Fatty acid metabolism in freshwater fish with particular reference to polyunsaturated fatty acids”	Archives of Animal Nutrition	UK	289	1.5	1996	Article
4	Ó Monroig, DR Tocher, JC Navarro [33]	“Biosynthesis of polyunsaturated fatty acids in marine invertebrates: Recent advances in molecular mechanisms”	Marine Drugs	Spain	266	5.4	2013	Review
5	Y Li, O Monroig, L Zhang et al. [34]	“Vertebrate fatty acyl desaturase with Δ4 activity”	PNAS	China	244	9.1	2010	Article
6	MJ Leaver, JM Bautista, BT Björnsson et al. [35]	“Towards fish lipid nutrigenomics: Current state and prospects for fin-fish aquaculture”	Reviews in Fisheries Science	UK	238	5.3	2008	Article
7	S Morais, O Monroig, X Zheng et al. [36]	“Highly unsaturated fatty acid synthesis in Atlantic salmon: Characterization of Elovl5- and Elovl2-like elongases”	Marine Biotechnology	UK	228	2.8	2009	Article
8	E David, A Tanguy, K Pichavant, D Moraga [37]	“Response of the Pacific oyster *Crassostrea gigas* to hypoxia exposure under experimental conditions”	FEBS Journal	France	204	4.2	2005	Article
9	DR Tocher, X Zheng, C Schlechtriem et al. [38]	“Highly unsaturated fatty acid synthesis in marine fish: Cloning, functional characterization, and nutritional regulation of fatty acyl Δ6 desaturase of Atlantic cod (*Gadus morhua* L.)”	Lipids	UK	200	1.6	2006	Article
10	X Zheng, BE Torstensen, DR Tocher et al. [39]	“Environmental and dietary influences on highly unsaturated fatty acid biosynthesis and expression of fatty acyl desaturase and elongase genes in the liver of Atlantic salmon (*Salmo salar*)”	BBA-Molecular and Cell Biology of Lipids	UK	189	3.3	2005	Article

**Table 3:** Top 10 highly cited authors with the greatest volume of documents in this field.

Authors	TD	TC	ACPD	TDO	TCO	h-Index	Affiliation (Current), Country
Tocher, D.R.	121	9145	76	406	33,572	95	University of Stirling, UK
Monroig, Ó.	91	4783	53	168	6778	45	CSIC—Instituto de Acuicultura de Torre de la Sal, Spain
Li, Y.	41	1510	37	146	4171	37	South China Agricultural University, China
Wang, S.	41	1448	35	125	3392	34	Shantou University, China
You, C.	36	1353	38	77	2381	29	Zhongkai University of Agriculture and Engineering, China
Navarro, J.C.	32	1106	35	163	6517	42	CSIC—Instituto de Acuicultura de Torre de la Sal, Spain
Dick, J.R.	26	2817	108	103	7916	48	University of Stirling, UK
Kabeya, N.	25	1054	42	55	1867	24	Tokyo University of Marine Science and Technology, Japan
Turchini, G.M.	25	1036	41	176	8465	49	Deakin University, Australia
Mai, K.	20	718	36	772	27,144	83	Ocean University of China, China

TD total documents, TC total citations, ACPD average citations per document, TDO total documents, and TCO total citations.

**Table 4:** List of the top 15 most productive journals.

Rank	Journal Name	TD	TD%	IF	h-Index	Categories
1	Aquaculture	107	9.88	3.9	40	Agricultural and Biological Sciences: Aquatic Science
2	Comparative Biochemistry and Physiology B	39	3.6	1.8	29	Biological and Agricultural Sciences: Aquatic Science
3	PLoS One	29	2.68	2.6	20	Multidisciplinary
4	Animals	27	2.49	2.7	9	Biological and Agricultural Sciences; Zoology and Animal Science
5	Lipids	27	2.49	1.6	20	Biochemistry, Genetics and Molecular Biology: Biochemistry
6	British Journal of Nutrition	24	2.22	3	19	Medicine (miscellaneous), Nutrition and Dietetics
7	BBA-Molecular and Cell Biology of Lipids	22	2.03	3.3	15	Genetics, Molecular Biology and Biochemistry.
8	Fish Physiology and Biochemistry	22	2.03	2.5	17	Aquatic Science, Molecular Biology, Biochemistry and Genetics: Physiology
9	Gene	20	1.85	2.4	12	Biochemistry, Molecular Biology and Genetics
10	Marine Biotechnology	20	1.85	2.8	15	Genetics, Biochemistry, and Molecular Biology
11	Aquaculture Nutrition	19	1.75	3.9	11	Aquaculture Nutrition
12	International Journal of Molecular Sciences	16	1.48	4.9	9	International Journal of Molecular Sciences
13	Journal of Agricultural and Food Chemistry	14	1.29	6.2	11	Journal of Agricultural and Food Chemistry
14	Aquaculture Reports	14	1.29	3.7	5	Aquaculture Reports
15	Scientific Reports	13	1.2	3.9	12	multidisciplinary

TD total documents, IF impact factor.

**Table 5:** Diversity and functional activities of desaturases in selected aquatic animals.

Species	Gene	Functional Activity/Key Finding	Reference
Grouper (*Epinephelus coioides*)	*Fads2*	Functions primarily as a Δ6 desaturase with minor Δ8 activity, indicating limited LC-PUFA biosynthetic capacity.	[64]
Chinese sturgeon (*Acipenser sinensis*)	*Fads2*	Fads2 exhibited Δ6 and Δ8 bifunctional desaturation activities and possessed capacities to convert 18:2n-6, 18:3n-3, 20:2n-6 and 20:3n-3 to 18:3n-6, 18:4n-3, 20:3n-6 and 20:4n-3, respectively.	[65]
Chinese sturgeon (*Acipenser sinensis*)	*Fads1*	Chinese sturgeon Fads1 exhibited Δ5 desaturation activity, efficiently converting 20:3n-6 to ARA and 20:4n-3 to EPA.	[66]
Common carp (*Cyprinus carpio*)	*Fads2a*, *Fads2b*	Fads2b demonstrated stronger ∆6 and ∆5 desaturase activities than Fads2a.	[67]
Dwarf surf clam (*Mulinia lateralis*)	*Fads a*, *Fads b*	Δ5 desaturation activity	[68]
European sea bass (*Dicentrarchus labrax* L.)	*Fad ∆6*	The sea bass FAD was more active towards 18:3n-3, with 14.5% being converted to 18:4n-3, compared to 5.6% of 18:2n-6 converted to 18:3n-6.	[69]
Eurasian perch (*Perca fluviatilis*)	*Fads2*	Showed Δ6 desaturation activity toward C18 PUFA substrates	[70]
Spotted scat (*Scatophagus argus*)	*Fads2*	Δ6 desaturation activity	[71]
Paiche (*Arapaima gigas*)	*Fads2*	Δ6 activity complemented with some capacity for Δ8 desaturation.	[72]
Chu’s croaker (*Nibea coibor*)	*Fad ∆6*	The Chu’s croaker desaturase was a Fad2 with ∆6 Fad activity, with 9.36% conversion of 18:2n-6 to 18:3n-6 and 4.93% of 18:3n-3 to 18:4n-3	[73].
Rainbow trout (*Oncorhynchus mykiss*)	*Fads2b*	Δ5 and Δ6 desaturase activities	[74]

**Table 6:** Functional activities and substrate specificities of Elovl enzymes involved in LC-PUFA biosynthesis in selected fish species.

Species	Gene	Functional Activity/Key Finding	Reference
Chinese sturgeon (*Acipenser sinensis*)	*Elovl2*	Efficiently elongates C20 (ARA and EPA) and C22 (22:4n-6 and 22:5n-3) substrates	[78]
Silver barb (*Puntius gonionotus*)	*Elovl5*	Molecular characterization, tissue distribution, and nutritional regulation of the *Elovl5* gene were investigated.	[79]
Tambaqui (*Colossoma macropomum*)	*Elovl4a*, *Elovl4b*	Both Elovl4 enzymes have activity toward all the PUFA substrates assayed (18:3n-3, 18:2n-6, 18:4n-3, 18:3n-6, 20:5n-3, 20:4n-6, 22:5n-3, 22:4n-6 and 22:6n-3)	[80]
Orange-spotted grouper (*Epinephelus coioides*)	*Elovl5*	Effectively elongates both C18 (18:2n-6, 18:3n-3, 18:3n-6 and 18:4n-3) and C20 (20:4n-6 and 20:5n-3) PUFAs, but not the C22 substrates.	[81]
African catfish (*Clarias gariepinus*)	*Elovl4a*, *Elovl4b*	Both Elovl4 enzymes have activity towards all the PUFA substrates assayed (18:4n-3, 18:3n-6, 20:5n-3, 20:4n-6, 22:5n-3, 22:4n-6 and 22:6n-3), producing elongated products of up to C36	[82]
Large yellow croaker (*Larimichthys crocea*)	*Elovl4*, *Elovl5*	Elovl4 could effectively convert both C20 and C22 PUFA to longer polyenoic products up to C34, while Elovl5 displayed high elongation activity towards C18 and C20 PUFA, with only low activity towards C22 PUFA	[83]
Black seabream (*Acanthopagrus schlegelii*)	*Elovl4a*, *Elovl4b*	Both Elovl4a and Elovl4b proteins had the ability to elongate 20:5n-3 and 22:5n-3 to 24:5n-3.	[84]
Rabbitfish (*Siganus canaliculatus*)	*Elovl8a*, *Elovl8b*	Elovl8b could elongate C18 (18:2n-6, 18:3n-3 and 18:4n-3) and C20 (20:4n-6 and 20:5n-3) (PUFAs) to (LC-PUFAs) whereas Elovl8a lacked this ability.	[85]
Rainbow trout (*Oncorhynchus mykiss*)	*Elovl4b1*, *Elovl4b2*	Elovl4b1 and Elovl4b2 can effectively convert C18–22 (PUFAs) to the polyenoic products up to C32 or C36. Especially, compared with Elovl4b2, Elovl4b1 had much higher efficiency for converting 20:5n-3 and 22:5n-3 to 24:5n-3.	[86]
Orange-spotted grouper (*Epinephelus coioides*)	*Elovl4*	Elovl4 elongates saturated (especially C24:0 and C26:0) and C20–C22 polyunsaturated FAs into long-chain products up to C36.	[87]
Golden pompano (*Trachinotus ovatus*)	*Elovl4b*	Elovl4b could efficiently elongate 18:2n-6, 18:3n-6 and 20:5n-3 FAs up to 20:2n-6, 20:3n-6 and 22:5n-3, respectively.	[88]

## Data Availability

No new data were created or analyzed in this study. Data sharing is not applicable to this article.
